# Antiviral defence arsenal across members of the *Bacillus cereus* group

**DOI:** 10.1038/s41598-025-86748-8

**Published:** 2025-02-10

**Authors:** Elise July, Annika Gillis

**Affiliations:** Laboratory of Food and Environmental Microbiology, Earth and Life Institute, Université Catholique de Louvain, Croix du Sud 2, Box L7.05.12, 1348 Louvain-la-Neuve, Belgium

**Keywords:** *Bacillus cereus* group, Bacteriophages, Phage defence systems, Defence systems association, Defence systems, Genomic location, Bacteriophages, Comparative genomics, Mobile elements, Data mining

## Abstract

Bacteria co-evolve with bacteriophages to overcome each other’s defence arsenal. *Bacillus cereus* group gathers bacteria of medical and agricultural importance, including foodborne pathogens. So far, few studies have portrayed a complete defence arsenal of microorganisms, and the role of antiviral systems in the *Bacillus cereus* group has been overlooked. Here, we investigate the repertoire of defence systems in 6354 *B. cereus* group’s genomic assemblies, using bioinformatics tools DefenseFinder and PADLOC. Our analyses provide an overview of the diversity and abundance of defence systems in this group, with 83,738 systems distributed by 2 to 33 within each assembly. Comparing PADLOC and DefenseFinder predictions showed that the most prevalent strategy is Restriction-Modification, but many abortive infection systems also intervene in the group’s defence, such as Septu, Gabija and Lamassu. Most defences were encoded on both plasmids and the chromosome, though some tend to have a preferential genomic location. We also studied the defence systems associations within the genomic assemblies. Overall, our results establish a baseline picturing the rich and complex antiviral arsenal encoded by *B. cereus* group’s species and provide clues for studying co-existing strategies displayed by these bacteria to subvert phages and other MGEs invasions.

## Introduction

Bacteria co-evolve with bacteriophages (phages), interlocked in a continuous arms race to overcome each other’s defence arsenal. Multiple antiviral defence systems are typically encoded in prokaryotic genomes, clustered within defence islands (DIs) and often associated with mobile genetic elements (MGEs) usually integrated into distinct hotspots^1,2^. Defence genes can make up 10% of the total prokaryote genome and have an extremely dynamic turnover^3^. Such gain and loss is frequently due to the high rate of horizontal gene transfer (HGT)^4^ and mediated by MGEs such as conjugative elements, transposons, prophages and phage satellites. In fact, MGEs carry a significant fraction of the repertoire of cellular antiviral defence genes^2^, leading to a high variability in the defence arsenal observed even among closely related bacterial strains^3–5^. Antiviral defence systems seem to act as a community resource, shared between closely related strains via MGEs^4^ and participating in inter-MGEs warfare by not only defending the host bacterium against invading phages, but also by protecting resident MGEs against invading ones^2^.

Upon phage invasion, two defensive outcomes can possibly emerge: the “altruistic” suicide of the host bacterium or the survival of the cell through the activation of defence mechanisms. Abortive infection (abi) strategies, or “altruistic” cell suicides, play an important role in prokaryotic defence. Their mechanisms are based on the activity of a range of single proteins to more complex multi-protein systems. In parallel, Restriction-Modification (RM) (innate immunity) is one of the most prevalent mechanisms in prokaryotes. RM systems are present in most bacterial genera and discriminate between *self* and *non-self* DNA via methylation of host DNA, before relying on restriction nucleases for exogenous DNA degradation^6^. On the other hand, adaptive immunity is mainly due to CRISPR-Cas systems that “keep in memory” information on previous phages encounters in the form of small RNAs^7^. Recent studies have uncovered dozens of defence systems, among which many have no known mechanism of action yet. Still, it has become apparent that prokaryotic immunity exhibits conserved molecular features, for instance: TIR domain signalling (*i.e.* CBASS, Pycsar, Thoeris, Rst_TIR-NLR)^8^, SMC domains (*i.e*. Lamassu, Wadjet)^9,10^, cGAS–STING pathways (*i.e.* CBASS, Pycsar)^11,12^ , STAND-NLR modules (*i.e*. Rst_TIR-NLR, AVAST, bNACHT)^13–15^, HEPN domains (*i.e.* Gao_ApeA, PD-Lambda-5, abi proteins such as AbiA, AbiJ or AbiV, PrrC, RloC and CRISPR-Cas)^14,16^, OLD nucleases (*i.e.* Gabija, PARIS, Septu)^17^ and many more^9,10,14,15,18^.

The *Bacillus cereus* group, also known as *B. cereus sensu* *lato* (*s.l.*), gathers common contaminants in the agro-food industry, as well as species with medical and biotechnological importance. Ubiquitous and sporulating, some pathogens of this group are responsible for gastrointestinal toxi-infections. Because members of this group have very conserved genomes, many species are distinguished by characters carried by MGEs, such as virulence factors and toxins. The wide variety of plasmids found in the *B. cereus* group includes various transmissible and conjugative plasmids with genome sizes ranging from 2 to 600 kb and several kinds of plasmidial prophages^19–22^. The taxonomy of this group has been subjected to many revisions, and to date about 20 species have been recognized, including *B. cereus sensu* *stricto* (*s.s*.) – vector of toxins (*i.e*. cereulide, CytK, hemolysin BL) responsible for emetic and diarrheal syndromes, *Bacillus thuringiensis* – vector of Cry toxins widely used as biopesticides, and *Bacillus anthracis* – the agent of anthrax disease^23,24^. Some other members of the group not formally belonging to these species are referred to as *B. cereus s.l*.

Although the *B. cereus* group is a good candidate to study phage-bacteria interactions and phage-based control measures^25,26^, there is a gap of knowledge regarding the role of antiviral systems in this group. Indeed, within the field of prokaryotic immunity, only a few studies have been conducted on depicting a complete defence arsenal of bacteria, except in model organisms such as *Escherichia coli*^27^, *Pseudomonas aeruginosa*^28^, and Actinobacteria^29^. In this work, we investigate the repertoire of defence systems in the *B. cereus* group pan-genome. By analysing over 6300 genomic assemblies belonging to *B. cereus s.l*., we demonstrate the diversity of defence systems and their respective importance within this bacterial group, as well as their genomic location (*i.e.* chromosome *vs*. plasmids), and their tendency to co-exist in a single strain. Our analysis represents a repository of the defence systems in *B. cereus* species that may serve as a resource for the study of co-existing strategies displayed by this bacterial group to subvert phages’ attacks and, possibly, other MGEs’ invasions.

## Methods

### Genomic database

The list of all 6366 genomic assemblies from the *B. cereus* group (NCBI Taxonomy ID N° 86661) used in this work is available in Supplementary Data 1. The genomic assemblies were downloaded from the NCBI database (March 2024) and comprise assembly levels ranging from contigs (n = 2534), scaffolds (n = 3261), chromosomes (n = 105), to complete genomes (n = 466). As there are more fragmented assemblies than complete genomes for the *B. cereus* group in the NCBI database, both complete genomes and fragmented assemblies were included in the analyses to broaden the scope of this study in capturing the diversity of antiviral defence systems across the entire bacterial group. Out of the assemblies obtained from the NCBI database, 27 species from the *B. cereus* group were identified and 12 assemblies belonging to *Bacillus* sp. and *Bacillus clarus* were removed as these do not belong to *B. cereus* group or are not validly published as an accepted species under the International Code of Nomenclature of Prokaryotes. The group referred to as “Other”, encompassing less than 10 genomic assemblies each, comprises *B. cereus s.l.* (unspecified species of the group), *Bacillus bombysepticus*, *Bacillus shihchuchen, Bacillus gaemokensis, Bacillus hominis, Bacillus luti, Bacillus manliponensis*, *Bacillus paramobilis*, *Bacillus proteolyticus*, *Bacillus sanguinis* and *Bacillus dicomae* species.

### Detection of defence systems

Defence systems were predicted by DefenseFinder^30^ (with models v1.2.3) and PADLOC v2.0.0^31,32^, using default parameters. A list of all defence systems detected in this work is available in Supplementary Data 2 and 3 (DefenseFinder and PADLOC, respectively). These bioinformatics tools compiled a total of 100,322 systems. Because some systems are classified quite differently between DefenseFinder and PADLOC, systems were re-named for comparison purposes, under a harmonised type and subtype (Supplementary Data 4). For instance, the PALOC “DRT_class_I” system denomination encompassed systems AbiA, AbiK, and AbiP2 while these abi systems were detected as separate entities by DefenseFinder. As a result, all these systems were given the attribution type DRT, subtype DRT_Class1_AbiAKP2. Overall, the DRT classes have been “erased” because no equivalence could be drawn between DefenseFinder and PADLOC classes and types of DRT systems. Similarly, the DefenseFinder “Abi2” captures AbiD, AbiD1 and AbiF systems whereas PADLOC “AbiD” encompasses AbiD, AbiD1 and AbiF. As a result, all these defence systems occurrences were attributed, in this work, to the novel type and subtype Abi2DF.

Additionally, the PADLOC generated dataset was cleaned up of some artefacts. In fact, Retron_XII proteins found in the dataset were systematically duplicated as Retron_I_C within the same genomes, at the same location, for the same protein. Given that a single protein cannot be two systems at the same time and because all these seven occurrences were identified by DefenseFinder as Retron_I_C, the Retron_XII duplicates were deleted from the PADLOC dataset. Other 382 PADLOC duplicate artefacts were identified and inspected individually. Most of these duplicates were related to unpublished candidate systems (PDC-x) from Payne et al*.* (2021)^33^. The PDC-x systems whose duplicate partner was a published system were deleted from the PADLOC dataset. It was the case for 343 pairs: SoFIC (97) and PDC-S04 (97), Septu (244) and PDC-M62 (203)/PDC-M01 (41), Dodola (1) and PDC-M37 (1), PDC-M46 (1) and Lamassu (1). The remaining 39 pairs of duplicates were not discriminated and should be considered carefully when studying the abundance and relative prevalence of each system in this dataset. Therefore, the total of systems detected in this work by PADLOC can be considered as slightly overestimated by 39 systems: ShosTA (2) and PsyrTA (2), CBASS_I (33) and Pycsar (33), DMS_other (2) and DRT_Class1_AbiAKP2 (2), PDC-M62 (2) and PDC-M01 (2).

### Validation of defence systems between PADLOC and DefenseFinder

#### Identical defence systems

A scoring scheme was established in order to compare whether the defence systems were detected by one or both tools: PADLOC = -1, DefenseFinder = 1, both = 0. Systems with a score = 0 were considered as identical systems if they met the following criteria, within single genomic assemblies: identical system type, identical protein(s) and identical count of protein(s). These identical systems were considered as validated matches between PADLOC and DefenseFinder.

A total of 509 defence systems were composed of the same sets of proteins but given a different system assignation by PADLOC and DefenseFinder (Supplementary Data 5). Most of these matches (486 pairs) related to unpublished candidate systems from Payne et al*.* (2021) (PDC-M60, PDC-M37, PDC-S28, PDC-M39, PDC-M46, PDC-S06, PDC-M01, PDC-M62) or to DMS_other systems, which is a loose PADLOC model that catches many different DNA-modification systems including BREX and DISARM^33^. These 486 pairs were considered as identical systems and validated, with their novel type and subtype those attributed by DefenseFinder. Furthermore, Dynamins and Eleos (described as a Dynamins-like defence system) were often found as couples and were thus grouped together as a same system type, Dynamins. In some rare occasions, the same sets of proteins were attributed to either PsyrTA and ShosTA (1), Pycsar and CBASS_I (10), Stk2 and PD-T4-6 (8), AbiO and Nhi (4). These were not considered as matches, to avoid having to choose between either systems types. We can thus consider that the number of validated systems is slightly underestimated.

#### Similar defence systems

The similarity of defence systems composed of different genes count and/or proteins was also evaluated, by using a TRUE/FALSE scheme. Notwithstanding the systems possessing all identical proteins, we found approximately 2500 systems that partially matched between PADLOC and DefenseFinder datasets, by sharing some degree of protein similarity (different systems type and/or count of proteins and/or identity of proteins) (Supplementary Data 6). To avoid the arbitrary process of choosing the key proteins belonging or not to each system, these possible matches were considered as different systems.

At the end of the validation process, we have validated 16,584 systems, identical between DefenseFinder and PADLOC, within 5683 genomic assemblies (Supplementary Data 7). The full dataset of systems (cleaned up of duplicates) amounts to 83,738 different defence systems, in 6354 genomic assemblies (Supplementary Data 8).

### Proportion of genomes occupied by defence systems

The proportion of the bacterial genomes occupied by the detected defence systems per genomic assembly was calculated as the sum of all defence genes’ length in bp compared to the size of the full genomic assembly. These proportions were calculated for validated defence systems only.

### Defence systems association within genomic assemblies

A correlation matrix was used to calculate the tendency of defence systems to be encoded in the same genomic assemblies or not. The Spearman correlation factor between systems was calculated with a *p*-value of 0.05. The correlation was supported by Non-metric MultiDimensional Scaling (NMDS) analysis, based on a Bray–Curtis distance matrix. A Bray Curtis distance matrix was constructed to represent the presence/absence relationship between systems per assemblies. The NMDS analysis generated scores to interpret the distances and grouped the genomes based on their closeness.

### Comparison of the abundance of defence systems

Following Georjon et al. (2023)^29^, we used frequency and abundance to characterise the defence systems importance in the *B. cereus* group. But here, frequency was represented as the proportion of total occurrences of a system compared to the total of detected systems. Abundance was represented as the proportion of genomic assemblies encoding a system to the total of genomic assemblies. By this distinction, the diversity of a system in a species was represented while considering that some genomes encode several copies of the same system.

The prevalence of systems in the *B. cereus* group compared to the abundance in Bacillota (formerly Firmicutes) was calculated using Eq. 1, as:

1 $${\text{Estimation }}_{\text{over}/\text{under}-\text{representation}}= {\text{\% genome}}_{Bacillus\,cereus\,s.l.} - {\text{\% genome}}_{\text{Bacillota}}$$

The % genome_*Bacillus cereus s.l.*_ calculated by means of Eq. 2, represents the abundance of the defence system across the *B. cereus* group assemblies encoding (one or more) version of the system, calculated as the total count of genomic assemblies encoding the system divided by the number of genomic assemblies in the group (n = 6354):

2 $${\text{\% genome}}_{Bacillus\,cereus\,s. l. }= \frac{{count\,of\,strains}_{system}}{6354} *100$$

Abundance for validated defence systems was calculated with respect to a total of 6354 genomic assemblies rather than 5683, the number of assemblies where validated systems were found, in order to assess the entire *B. cereus* group.

The % genome_Bacillota_ is the number of complete genomes belonging to the class Bacillota encoding the defence system, as given by the DefenseFinder Webservice (09/09/2024)^34^.

### Figures and statistical analysis

Figures and statistical analyses were done using R v4.3.2., with support of AI. The R libraries that have been used for this work include data.table, dplyr, eulerr, ggbreak, ggplot2, ggrepel, Hmisc, hrbrthemes, packcircles, pheatmap, readr, readxl, stringr, tidyr, RColorBrewer, reshape2, scales, vegan, and viridis.

## Results

In this work, we used DefenseFinder v1.2.3^30^ and PADLOC v2.0.0^31^, to predict known defence systems encoded in a total of 6354 *B. cereus* group genomic assemblies sourced from the NCBI database. The level of these genomic assemblies ranged from contigs (n = 2529), scaffolds (n = 3260), chromosomes (n = 105), to complete genomes (n = 460). By including both complete genomes and fragmented assemblies, we have significantly increased the number of genomic assemblies per species belonging to the *B. cereus* group, expanding the representativeness of our dataset. Out of the species identified, some rare or unconfirmed species were grouped together as “Other” (see Methods), making *Bacillus albus* (n = 29), *B. anthracis* (n = 868), *B. cereus s.s.* (n = 2427), *Bacillus cytotoxicus* (n = 43), *Bacillus mobilis* (n = 44), *Bacillus mycoides* (n = 183), *Bacillus nitratireducens* (n = 15), *Bacillus pacificus* (n = 92), *Bacillus paramycoides* (n = 17), *Bacillus paranthracis* (n = 286), *Bacillus pseudomycoides* (n = 134), *B. thuringiensis* (n = 1321), *Bacillus toyonensis* (n = 341), *Bacillus tropicus* (n = 56) and *Bacillus wiedmannii* (n = 234) the main species in the studied dataset.

### Defence systems are lightweight, diverse and abundant across the *B. cereus* group

**Fig. 1:**
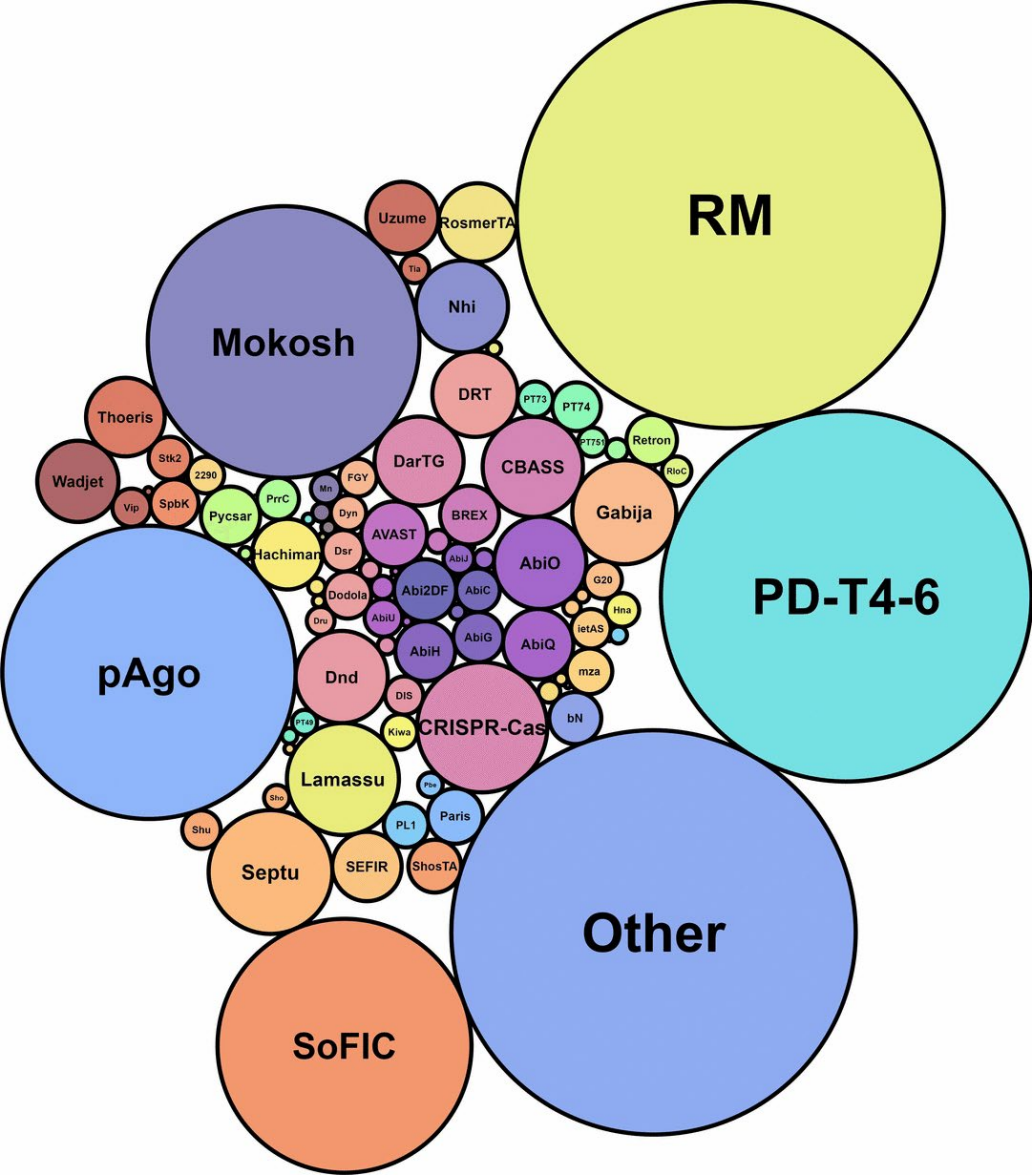
Representation of defence systems identified in the entire *Bacillus cereus* group. The most abundant systems are RM, PD-T4-6, Mokosh, pAgo, and PDC-x (other)^33^. Systems for which less than 50 occurrences in the *B. cereus* group have been counted are not labelled. Size of the circle represents the total number of predicted defence systems. Abbreviations: G20 (Gao_20), ietAS (Gao_ietAS), mza (Gao_mza), 2290 (Rst_HelicaseDUF2290), FGY (FS_GIY_YIG), Mn (Menshen), Vip (Viperins), Tia (Tiamat), Shu (Shedu), Sho (Shango), PL1 (PD-Lambda-1), Dyn (Dynamins), bN (NRL_bNACHT), PT73 (PD-T7-3), PT74 (PD-T7-4), PT751 (PD-T7-5_1), PT49 (PD-T4-9), DIS (DISARM), Dru (Druantia).

The analysis of defence systems, taking into account the results by DefenseFinder and PADLOC, predicted 100,322 systems in the *B. cereus* group’s genomic pool. After harmonisation of the detected systems under a common designation, and cleaning up of artefacts and duplicates (see Methods), a total of 83,738 individual systems remained and were considered to be part of the *B. cereus* group’s defence arsenal (Fig. 1). In total, we have identified 99 different types of systems (> 240 including subtypes) (Supplementary Data 8). Less than 10% of the systems (N = 5807) were encoded in complete genomes (n = 460) (Supplementary Fig. S1).

**Fig. 2:**
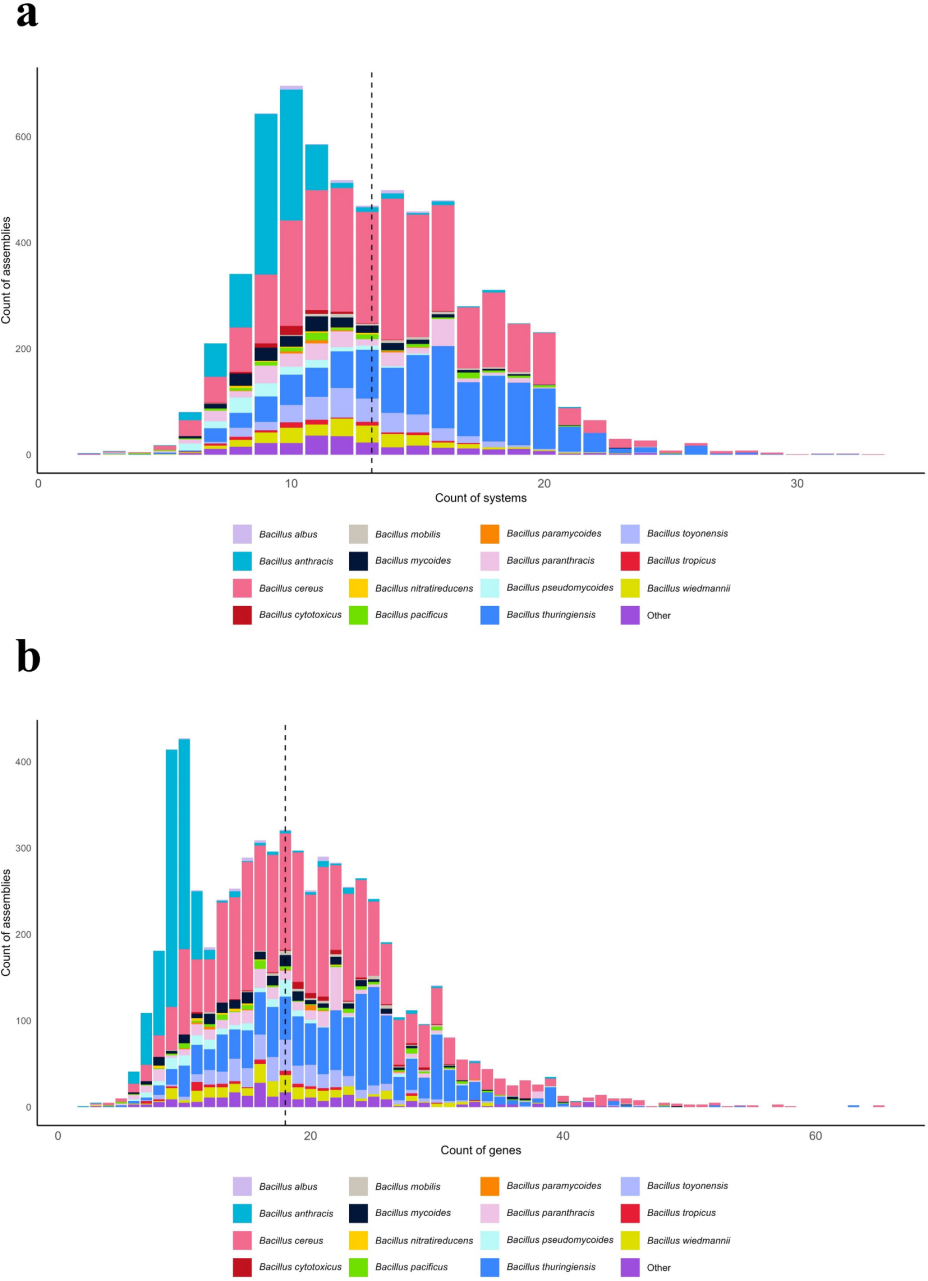
Distribution of defence systems and genes in the *Bacillus cereus* group. (**a**) Distribution of the total number of defence systems per genomic assembly (min = 2, max = 33, mean = 13, median = 13). Dashed line, average across the group. (**b**) Distribution of the total number of defence genes per genomic assembly (min = 2, max = 65, mean = 19, median = 18). Dashed line, average across the group**.**

Overall, most of the detected systems (75%) are composed of a single protein, or two proteins (14%). None of the tested genomic assemblies was devoid of defence systems. Genomic assemblies contain between 2 to 65 genes allocated to defence, with an average of 19, which make up for 2 to 33 systems (Fig. 2a,b). With 13 systems per assembly across the group (Fig. 2a) and 12.6 systems across complete genomes (Supplementary Fig. S2a-c), the *B. cereus* group average is quite higher than the average of other prokaryotes (five antiviral systems per genome, calculated based on complete genomes^30^).

**Fig. 3:**
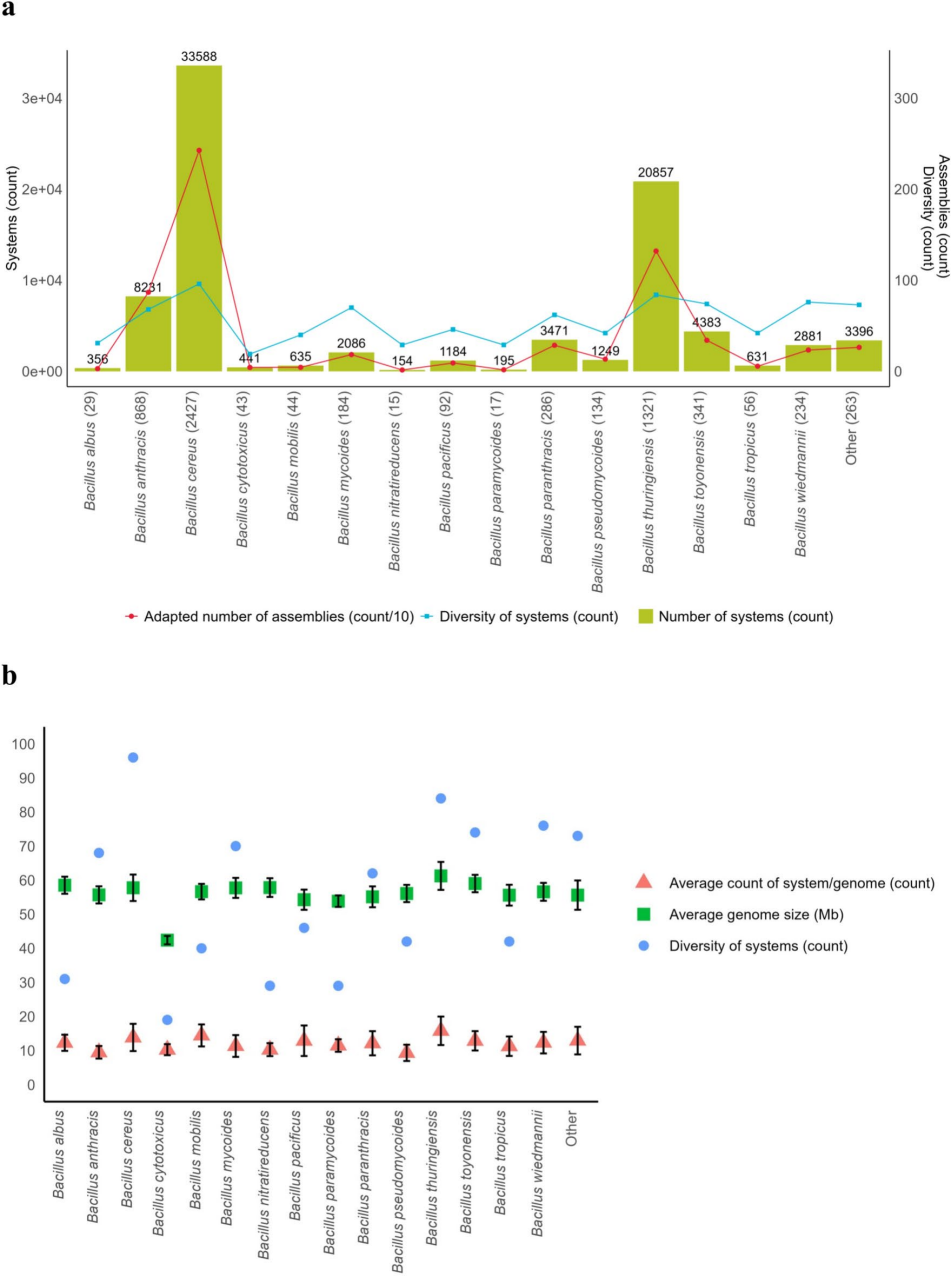
Repartition of defence systems detected in the *Bacillus cereus* group per species. (**a**) Abundance of systems per species in the *B. cereus* group. Species with the most genomic assemblies cumulate a higher number and diversity of systems. Overall, *Bacillus cereus sensu stricto* (*s.s.*) and *Bacillus thuringiensis* gather the highest number of systems for this bacterial group. Red line, count of assemblies divided by 10. Blue line, count of different types of defence systems. (**b**) Average number of defence systems and genomic assembly size per strain across species in the *B. cereus* group. For average genomic assembly size (in Mb) Y-axis should be divided by 10. Species with average largest genomic assemblies cumulate higher defence systems content. Across the group, genome size was ~ 5.8 Mb. Standard deviation calculated based on the number of genomic assemblies, for each species.

We also found a relationship between the size of the genomic assembly and the number of defence systems encoded, as the largest assemblies have higher defence systems content, except for *B. anthracis.* Assemblies of this latter species encode the least average number of defence genes and systems of the *B. cereus* group, although the level of diversity is within the range of the group (Fig. 3a,b). *B. cytotoxicus* also stands out with a particularly inferior average size of genomic assembly. Thus, the defence arsenal in *B. cytotoxicus* claims a higher proportion of the genome compared to other species of the group.

Looking at the distribution of defence systems across species, we found that defence content across the group mirrors the number of genomic assemblies available per each *B. cereus s.l*. species. The species with the richest defence arsenal are those with the highest number of assemblies analysed in this work. Indeed, out of 83,738 different defence systems predicted in the *B. cereus* group pan-genome, most of the systems were encoded in *B. cereus s.s*. (N = 33,588) and *B. thuringiensis* (N = 20,857) assemblies, which make up for most genomes in our dataset (Fig. 3a). Species encompassing a low portion of the genomic assemblies analysed gather fewer systems, *i.e. B. nitratireducens* (N = 154) and *B. paramycoides* (N = 195) (Fig. 3a, b). Also, a vast diversity in types of systems was observed across species. Given that the diversity of system types varies from less than 10 to more than 70, for an average genome size of 5.8 Mb, the level of diversity per species does appear to be related to the number of assemblies per species, rather than genome size (Fig. 3b). Indeed, the evolution of diversity of defence systems and the number of genomic assemblies for each species of the group follow similar trends (Fig. 3a).

### Defence systems’ abundance in the *B. cereus* group displays an important heterogeneity

Given the genomic closeness of the *B. cereus* group species, we have compiled the defence arsenal found among all *B. cereus* species available in the NCBI database, showing that RM and PD-T4-6 are by far the most prevalent defence systems in this bacterial group (Fig. 1). Our analysis reveals that close to every genomic assembly in our dataset encodes at least one RM system (99.8%), and one PD-T4-6 (99.7%) system (Supplementary Table S1). Individual assemblies most often encode several of these systems. For instance, most assemblies encode at least two RM, and up to nine RM systems are predicted in two *B. cereus s.s.* strains, with four RM_II, one RM_IIG, two RM_IV, one RM_I and one RM_III each. Most genomes also contain Mokosh (93.8%) and pAgo (92.1%). Many PDC-x^31^ (88.5%), SoFIC (55%), Septu (18.4%), CRISPR-Cas (16.3%), Lamassu (15.6%), Gabija (13.9%), Nhi (11.9%), DarTG (11.2%) and CBASS (10.7%) defence systems are also part of the *B. cereus* group arsenal. Frequencies for the remainder of predicted systems are much lower. For instance, 74 out of 99 systems are found in less than 5% of the assemblies each, and together account to 7.8% of systems in our dataset. The *B. cereus* group thus relies on a main set of strategies, with most of the systems’ diversity consisting of “rare” systems.

By comparing the abundance of the defence systems in the *B. cereus* group to their abundance in their phylum, we found that some of the frequencies are much higher than expected for Bacillota (Supplementary Fig. S3). But we also observed that some systems are less abundant than expected. In particular, CRISPR-Cas systems are encoded in 16.3% of genomic assemblies in the group, but in 34% of Bacillota complete genomes^34^.

Furthermore, by classifying systems based on the defence outcome (*i.e*. abi, possible abi, non-abi and unknown), our analysis revealed that the most represented defence outcome is cell death, encompassing 54.5% of defence systems in our dataset (Supplementary Fig. S4 and S5). Both (possible) abi and non-abi outcomes are represented within genomic assemblies analysed in this study, as all but 12 assemblies possessed at least one system of each.

### Validation of defence systems detected in the *B. cereus* group

The bioinformatics tools DefenseFinder^30^ and PADLOC^31^ have been developed to predict known prokaryotic antiviral defence systems within genomic sequences. Still, both programs work differently and provide different predictions (see Discussion). Therefore, to have a panorama of the antiviral defence systems encoded in the genome of members of the *B. cereus* group, it was first necessary to harmonise the different defence systems detected by both bioinformatics tools in our dataset (see Methods). Defence systems in both DefenseFinder and PADLOC datasets have been attributed a novel type and subtype (Supplementary Data 4), to harmonise the names of DefenseFinder and PADLOC systems and facilitate their comparison. A subset of systems detected by both DefenseFinder and PADLOC were matched by establishing a set of rules: identical systems were those that, within the same assembly, were composed of identical proteins and had the same number of genes. These DefenseFinder *vs*. PADLOC systems matches were considered a positive system identification, referred to as “validated systems” hereafter (Supplementary Fig. S6).

**Fig. 4:**
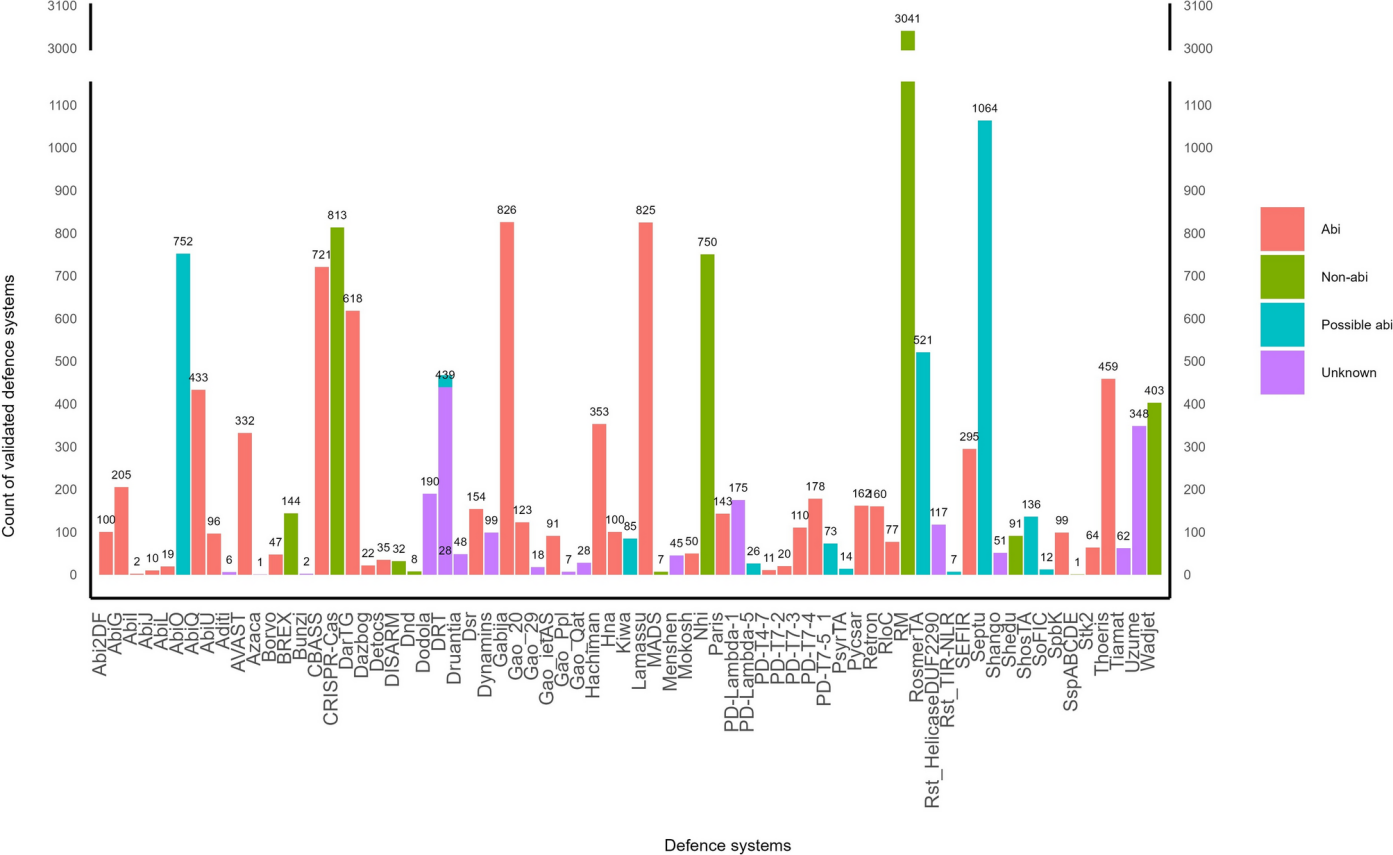
Total count of validated defence systems in the *Bacillus cereus* group. RM is the most frequent (14.3% of validated systems) and abundant (37.5% of genomic assemblies), followed by Septu (15.2% of frequency and 5.8% of abundance), Gabija (12.4% of frequency and 4.7% of abundance), Lamassu (12.1% of frequency and 4.6% of abundance), CRISPR-Cas (11.9% of frequency and 4.6% of abundance) and Nhi (11.8% of frequency and 4.5% of abundance). Most of the validated systems display abi outcome. Defence systems cell outcome indicated in colour: red, abi; green, non-abi; cyan, possible abi; mauve, unknown.

Out of the 83,738 defence systems detected in this work, 20% were identified by both DefenseFinder and PADLOC, and therefore identified as “validated systems”. These 16,584 validated systems possess the same type, number and identity of protein(s), within a single genomic assembly (Fig. 4 and Supplementary Data 7). These comprise 69 different types of systems across 5683 assemblies, which harbour between 1 and 15 systems (mean = 3, median = 2). Between 1 and 33 genes (mean = 6, median = 5) are allocated to defence, occupying between 0.01% and 0.99% (mean = 0.16%, median = 0.14%) of the genomic assemblies in the *B. cereus* group (Supplementary Fig. S7a-c).

### RM and abi are the two major defence strategies identified in the *B. cereus* group

The analysis of validated defence systems in this work highlighted that a total of 52 systems encoded in less than 5% of the genomic assemblies account for 23.3% of these validated systems (Supplementary Data 7). The most abundant defence systems in the group are RM, present in 37.5% of the genomic assemblies, followed by two (possible) abi systems, Septu (15.2%) and Gabija (12.4%) (Fig. 4). RM is an important antiviral strategy based on the recognition and destruction of *non-self* invading DNA. This defence strategy requires one to three genes for complete endonuclease and methyltransferase enzymatic activities, and is divided into four major types (type I to IV)^6^, which are all present in the validated defence arsenal of the *B. cereus* group identified in this work. In fact, a total of 1387 RM type II(G), 1288 RM type I, 191 RM type III and 175 RM type IV have been detected (Supplementary Data 7).

Interestingly, the top three of validated defence systems (Fig. 4) does not coincide with the ranking of most prevalent systems found in the full dataset (Fig. 1), due to detection differences between PADLOC and DefenseFinder. Out of the 83,738 systems detected across all the *B. cereus* group genomic assemblies, RM and PD-T4-6 are both found equally numerous, in almost all assemblies (99.8% and 99.7%, respectively). Regarding PD-T4-6, this type of system was detected only by PADLOC. The discrepancies in Mokosh defence system abundance before and after validation can also be explained this way: the majority of Mokosh systems are in fact Mokosh type II, of which none but one were detected by DefenseFinder. Same is valid for the SoFIC defence system. No argonautes proteins were detected by DefenseFinder in the dataset used for this work.

Nevertheless, our analysis validated that cell death is the most frequent defence outcome, with 9658 (possible) abi systems validated (58.2% of the defence systems) (Supplementary Fig. S8). Nine systems make up for the *B. cereus* group’s cell survival defence (non-abi) arsenal, composed of 5290 systems (*i.e.* BREX, CRISPR-Cas, DISARM, Dnd, MADS, Nhi, RM, Shedu and Wadjet; 31.9% of validated systems). Looking at the abundance of both outcomes, (possible) abi and non-abi systems are found in 66.3% and 63.2% of the genomic assemblies, respectively, indicating that most cells possess a multi-strategies defence arsenal.

### Defence systems Septu, AbiO, Nhi and DarTG are over-represented in the *B. cereus* group compared to other members of the phylum Bacillota

**Fig. 5:**
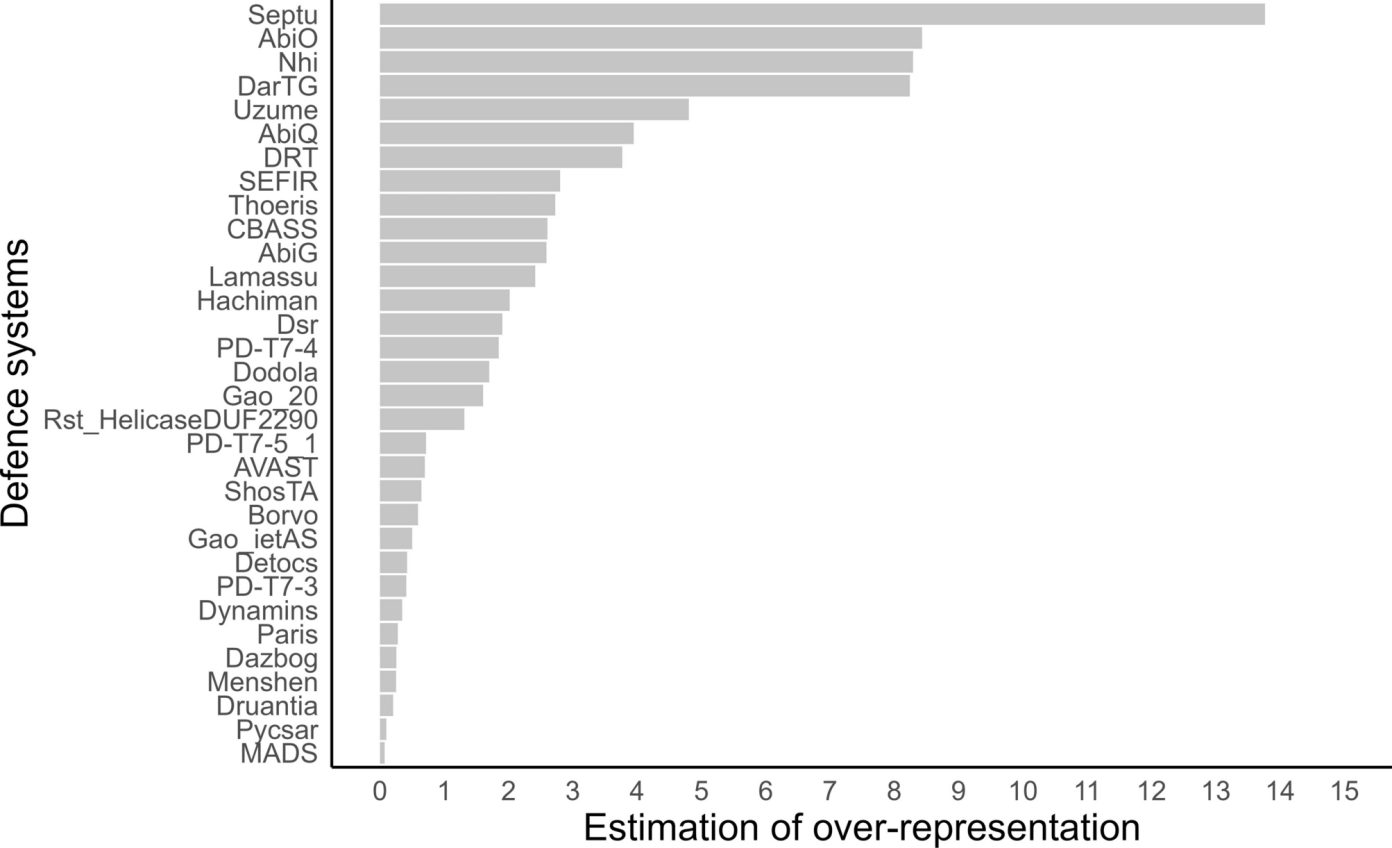
Comparison of abundance of validated defence systems between the *Bacillus cereus* group and phylum Bacillota. The estimation of under- or over-representation of defence systems encoded in the *B. cereus* group compared to systems encoded in Bacillota is calculated as the difference between the abundance in the *B. cereus* group genomic assemblies and the abundance in Bacillota complete genomes, as provided by DefenseFinder Webservice^34^ (see Methods, Eq. 1 and Supplementary Fig. S9 (for complete figure)).

The prevalence of some defence systems in our dataset being quite higher than in general prokaryotes, we have set out to compare the relative importance of each system in the group towards the abundance observed in phylum Bacillota^34^, in an effort to measure how divergent the *B. cereus* group could be within its phylum. The frequency of defence systems was significantly reduced during validation, with only 20% of detected systems that were identified by both PADLOC and DefenseFinder. Considering the possible bias of under-representing systems in our dataset due to the data skimming at validation step, we focused only on positive relationships between the *B. cereus* group and phylum Bacillota. Positive relationships mean that systems are over-represented in the group compared to the abundance in the phylum (Fig. 5 and Supplementary Fig. S9 (for complete figure)).

Our analysis shows that within the *B. cereus* group, Septu, AbiO, Nhi and DarTG systems exceed Bacillota phylum’s abundance by 13.8%, 8.4%, 8.3% and 8.2%, respectively.

### Within the *B. cereus* group members, defence systems are encoded with a few inter-species differences

**Fig. 6:**
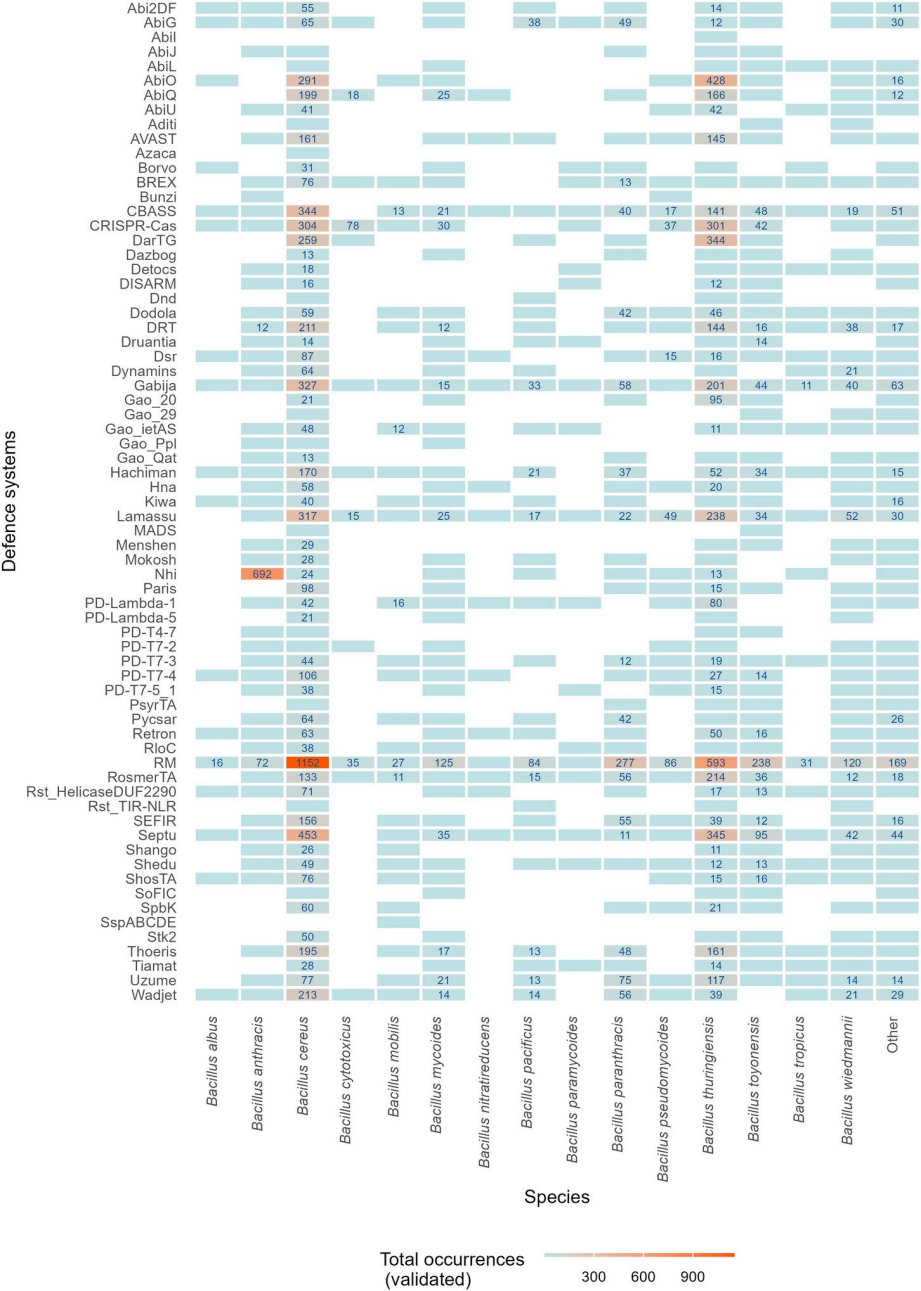
Overview of validated defence systems per species in the *Bacillus cereus* group. Except for Nhi and rare systems (< 10 occurrences), *Bacillus cereus *sensu stricto (*s.s.*) encompasses the highest frequency of the detected defence systems, with the exception of *Bacillus thuringiensis* for systems AbiO, DarTG, Gao_20, PD-Lambda-1, RosmerTA and Uzume. The most abundant species, *B. cereus s.s.* and *B. thuringiensis* possess the most numerous and diverse systems. The colour scale represents the total count of validated defence systems. These counts are displayed in boxes, shown only for > 10 occurrences.

Consistent with the trend observed in non-validated defence systems in our dataset, the species gathering the most genomic assemblies, *i.e. B. cereus s.s.* and *B. thuringiensis*, cumulate the highest quantity and diversity of validated systems (Figs. 3a,b, 6). *B. cereus s.s.* and *B. thuringiensis* sum up the highest frequency of defence systems for a vast majority of systems. However, there are few exceptions for rare systems (< 10 occurrence per species) and the Nhi defence system. Out of the 750 validated Nhi systems, 92.3% are encoded in *B. anthracis* assemblies. Interestingly, looking at the abundance of CRISPR-Cas within species, our results indicate that this type of system is close to absent in *B. anthracis*, with 0.3% of assemblies containing a CRISPR-Cas system, while 97.7% of* B. cytotoxicus* genomes encode at least one CRISPR-Cas system (Supplementary Table S2). The abundance of this defence system within *B. pseudomycoides* and *B. thuringiensis* is closer to the average of prokaryotes (*i.e*. 39%)^30^, but the remaining species of the *B. cereus* group analysed in this work, including *B. cereus s.s.*, do not exceed 13.6% of systems’ abundance.

### Defence systems DarTG, AbiO, and CRISPR-Cas display strong association in the *B. cereus* group

**Fig. 7:**
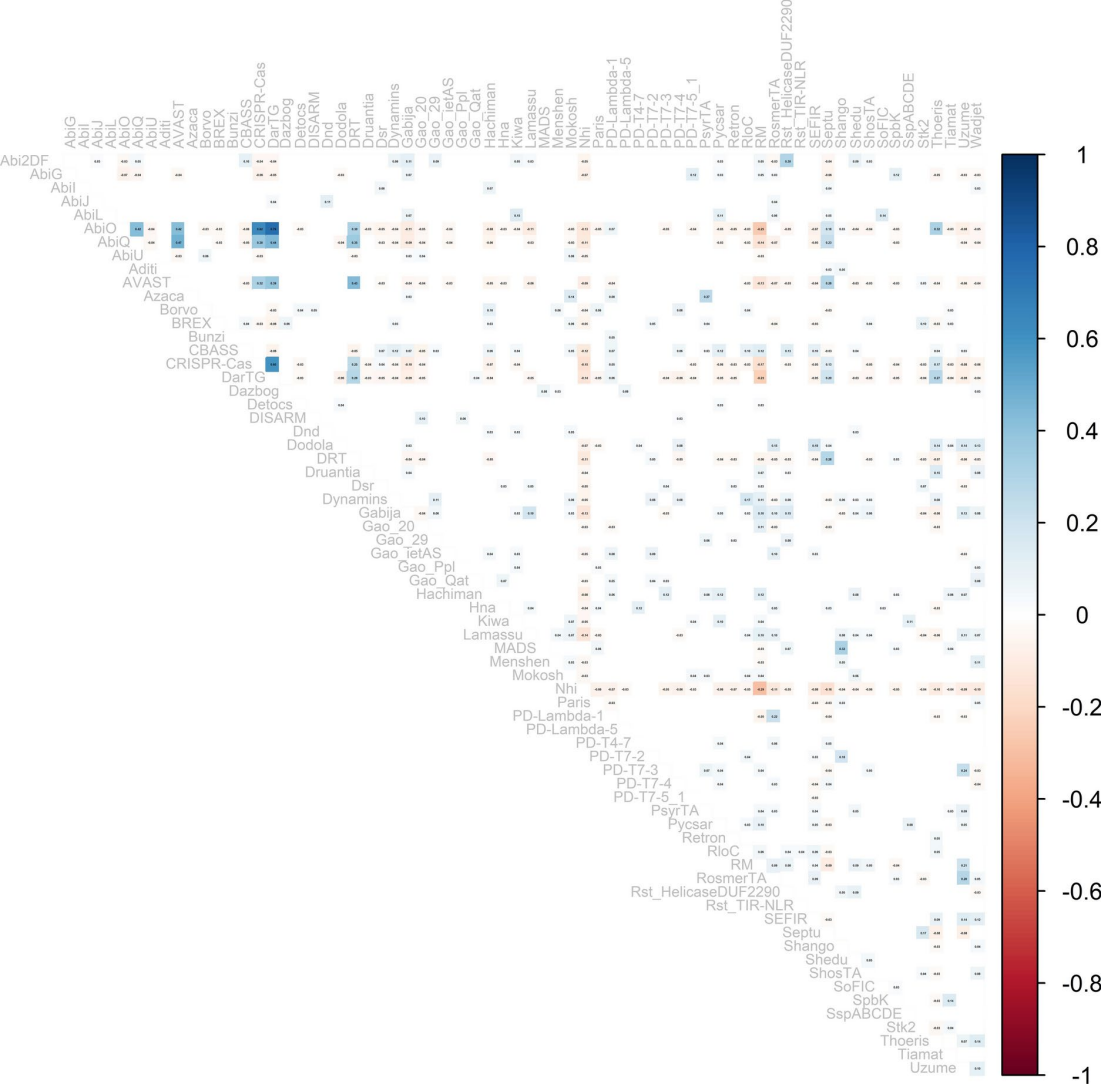
Significant correlations between validated defence systems in the *Bacillus cereus* group. Spearman correlation factors (*p*-value > 0.05) are indicated in the boxes. In blue, positive correlation; in red, negative correlation.

In an attempt to assess whether defence systems have a tendency to be encoded together or be negatively associated within genomic assemblies in the *B. cereus* group, a Spearman correlation analysis (*p*-value > 0.05) was carried out in this work. The results indicate that DarTG, AbiO, and CRISPR-Cas present a strong positive correlation to each other, suggesting that these systems may often be encoded together in members of the *B. cereus* group, coupling both abi (AbiO and DarTG) and non-abi (CRISPR-Cas) defence outcome strategies (Fig. 7). These three systems are also moderately correlated to DRT, AbiQ and AVAST. Interestingly, there was a slight negative association between the systems mentioned above (AbiO, AbiQ, AVAST, DarTG and CRISPR-Cas) and RM systems. Our results also show that Nhi is negatively correlated to most other systems, in particular RM systems (Fig. 7). As Nhi is mostly observed in *B. anthracis* genomes (Fig. 6 and Supplementary Fig. S5), it seems Nhi plays an important role in this species. Most other relationships detected between systems in our analysis display weak correlations. The isolation of Nhi from other defence strategies, as well as the opposition between RM and AVAST, AbiQ, DarTG, CRISPR-Cas and AbiO was verified by Non-metric MultiDimensional Scaling (NMDS) analysis (Supplementary Fig. S10).

### Defence systems in the *B. cereus* group are equally distributed on plasmids and chromosome

**Fig. 8:**
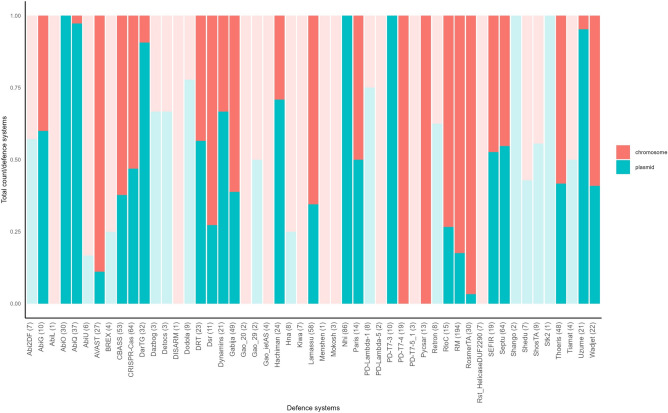
Validated defence systems encoded in the chromosome and plasmids in the *Bacillus cereus* group. Only complete genomes. Most defence systems are present on both genomic elements. Repartition expressed as a proportion between 0 and 1. Occurrences per system in parenthesis. Red, chromosome; blue, plasmid. Light red and blue colours indicate systems with < 10 occurrences.

Knowing that the *B. cereus* group species are characterised by their plasmidial content, we decided to investigate the repartition of validated defence systems on the plasmids *vs*. chromosome of complete genomes. A total of 1106 validated defence systems were found in 395 complete genomes of the *B. cereus* group. In this reduced dataset, (possible) abi strategies represent the majority of systems (54.1%), while non-abi strategies encompass 34.2% of the detected systems. Our results indicate that defence systems are found almost equally distributed on both plasmids (47%) and the chromosome (52.3%) (Fig. 8). However, some defence systems are found preferentially onto either plasmids or the chromosome. For instance, all Pycsar, PD-T7-4 and most RM, RloC, RosmerTA and AVAST systems are encoded on the chromosome. In contrast, all Nhi, PD-T7-3, AbiO and most AbiQ, DarTG and Uzume are encoded on plasmids.

### The case of defence system Nhi in *B. anthracis*

The validated defence arsenal within *B. anthracis* is composed in the vast majority by Nhi systems (692 out of 908). Nhi is often the only validated system in *B. anthracis* genomic assemblies due to the validation rules applied in this work (see Methods). Nhi has recently been described as a single nuclease-helicase enzyme, SERP2475, providing antiviral protection without causing abortive infection^35^. The RM systems are also present in a few *B. anthracis* genomic assemblies and other systems are rarer. Based on these observations, the presence of Nhi seems to be isolated from the presence of other systems, which is also indicated by our correlations results (Fig. 7). Furthermore, analysing only complete *B. anthracis* genomes, we examined the genomic repartition of the 92 identified defence systems. Out of these, 81 were Nhi systems, all encoded on plasmids, mostly pXO1 virulence plasmid (68 out of 81). Looking closely at pXO1 from *B. anthracis* Sterne, a large 181 kb plasmid encoding the anthrax toxin genes (GenBank N° AF065404), we found that Nhi protein is annotated as *pXO1-133* gene, protein AAD32437.1^36^. This *pXO1-133* gene was one of the 70% ORFs on pXO1 which did not have significant similarity to sequences available in open databases described by Okinaka et al*.* (1999)^36^. This gene is located outside of the plasmid pathogenicity island, next to *pXO1-132*, coding for a phage XerS integrase (protein AF065141).

## Discussion

### Database bias

Due to their medical and agronomical importance, strains of *B. cereus s.s., B. anthracis* and *B. thuringiensis* have been extensively sequenced, more than other members of the *B. cereus* group. Therefore, the database used for the analysis in this work is biased towards these three members of the group. Also, our analysis relies not only on complete genomic assemblies, but also on fragmented (partial) genomes, which can impact the prediction of potential defence systems at the fragments’ extremities. As a result, some defence systems encoded within fragmented genomic assemblies may remain undetected, and the number and diversity of the presented arsenal of defence systems in the *B. cereus* group should be considered as underestimated. Still, we have observed similar trends when comparing our dataset of 460 closed genomes to the full 6354 dataset, including fragmented assemblies. For instance, the 5807 systems identified in 460 complete genomes represent 6.9% of the 83,738 systems identified and 7.2% of the assemblies analysed in this work.

### Taxonomy bias

Due to the genomic proximity of *B. cereus s.l.* species, most are differentiated by traits carried by MGEs, ensuing a complicated and changing taxonomy. Therefore, members of the *B. cereus* group can be subjected to species misidentification in the database retrieved from NCBI to perform our analysis. This might influence the relative importance of defence systems per species in our analysis.

### Defence systems detection bias

As previously explained, the defence systems models databases and algorithms behind PADLOC and DefenseFinder tools are distinct, creating a detection bias. These tools rely on sequence homology analysis and comparison to Hidden Markov Models (HMMs), but both programs work differently. First, their defence system detection default models are different and do not include all the same systems. Then, their system validation process varies: while both tools rely on gene presence/absence and synteny criteria, DefenseFinder also relies on MacSyfinder^37^ rules to analyse the genomic context and validate the systems. The difference in the number of defence systems detected by each bioinformatics tool used in this work can also be attributed to the fact that both tools do not use the same databases. For instance, close to 15,000 systems detected by PADLOC in this work are unpublished candidate systems^31^ not included in the prediction models of DefenseFinder.

In this study we have harmonised defence systems’ names between PADLOC and DefenseFinder, which was quite straightforward for most systems, but presented challenges in some cases where grouping several systems together was found as a solution (see Methods). Therefore, some systems identifications are less detailed in this work than by PADLOC or DefenseFinder individually (*i.e*. some pooled abi and DRT systems). Also, in some instances, the same sets of proteins were given a different system assignation by PADLOC and DefenseFinder. In other cases, defence systems detected by PADLOC or DefenseFinder shared some but not all proteins. Additionally, it is well established that some proteins are shared between defence systems. Such systems overlaps were found in this work, for instance, for proteins sharing SMC-like domains (Lamassu and Wadjet), TIR-domains (SEFIR and Thoeris), PT modifications (Dnd and Pbe), or RM based systems (Dnd and RM_II) (Supplementary Data 6). Some defence systems associations such as PrrC and RM_I have already been described in previous studies, as PrrC is an effector dependent on the RM type I system^16^. Some PrrC systems predicted by DefenseFinder are nested within RM_I systems, predicted by PADLOC. In this work, we attempted to rule in or out some of these mismatches (see Methods), while trying to keep these interpretations to a minimum.

### Defence systems validation bias

In an effort to substantiate the defence systems detected in the *B. cereus* group, we have matched together identical systems detected by both PADLOC and DefenseFinder. At the end of this validation step, 20% of the systems, out of the 83,738 detected, remained. Therefore, absence of defence systems in this dataset is not an indication that systems are not present in this bacterial group. These validated results should rather be interpreted as a strong indication that the validated systems are present in the *B. cereus* group.

### The *B. cereus* group presents an abundant and diverse defence arsenal

Our analysis of DefenseFinder and PADLOC antiviral systems prediction is based on 6354 *B. cereus* group genomic assemblies. We found that most of the group’s defence arsenal is composed of small-sized systems (on or two proteins). A maximum of 65 genes and 33 systems have been found per singular genomic assembly, with an average of 13 systems per assembly, which is quite higher than the average of prokaryotes (five systems per genome). Both cell suicide and cell survival strategies were found in almost all assemblies, indicating the group relies on both defences to defeat phages’ attacks. The importance of the abi outcome in prokaryotes has already been highlighted in another work, predicting that 72.5% of prokaryotic genomes encode at least one abi system^38^, while RM are present in about 75% of microbial genomes^30^. In the *B. cereus* group, the primary defence strategies are RM and abi, present in at least 37.5% and 66.3% of assemblies, respectively, when considering only validated systems. Though RM is the single most frequent system in the group amounting to 19.5% of all detected systems, the arsenal of abi systems is much more diverse ranging from complex multi-protein systems such as Lamassu, to single abortive proteins such as PD-T4-6. The prevalence of defence systems such as Septu, Lamassu and Gabija was validated within the group, but other (possible) abi systems could play an even bigger part in the *B. cereus* group “altruistic” suicide arsenal: PD-T4-6, pAgo, Mokosh and SoFIC.

Gabija is a widespread abi defence system predicted to exist in about 8.5% of prokaryotes, composed of two proteins GajA and GajB^10^. These proteins operate as sensors and effectors for (d)A/(d)GTP hydrolysis, nucleotide depletion and DNA cleavage, causing a cascade suicide effect upon virulent phages infection^39^. Septu, a possible abi defence system, is made of two proteins PtuA, with ATPase domain, and PtuB, with HNH endonuclease domain and is predicted to exist in 4.1% of prokaryotic genomes^10^. These two proteins operate as a PtuAB complex of distinctive horseshoe-like configuration^10,40^.

Coincidently, the presence of few systems in the *B. cereus* group has already been detected by BLASTP analysis and domain searching by Zheng et al. (2020)^7^, *i.e.* RM, Septu, Lamassu, Gabija, Wadjet, Zorya, Druantia, Thoeris, Hachiman, Kiwa, Shedu and DISARM. These observations coincide with our results (Figs. 1,4), except for system Zorya, which was not part of our findings. We have also identified a significant number of unpublished candidate systems (PDC-x)^31^ in this bacterial group.

Species members of the *B. cereus* group were originally described as ecotypes distinguished by their plasmid content, in particular taking into account virulence factors and environmental adaptation traits. In the light of phylogenetic and genomic analyses, the group was divided into clades with multiple cross-overs between species^41^. As a result, the *B. cereus* group has undergone several taxonomic revisions over the years, creating a bed for ambiguous species definition. In this context, we sought out to identify whether defensive traits can be species-specific in this group. The prevalence of defence systems across species belonging to the *B. cereus* group was overall a reflection of the availability of genomic assemblies per species in our dataset. Still, few species-specific trends can be highlighted. For instance, a significant prevalence of Nhi was validated in *B. anthracis*, which displays an otherwise similar defence arsenal to other members of the group. Though Nhi (SERP2475 and its homologs) are likely disseminated through HGT and Nhi is exclusively present on plasmids in our dataset of complete genomes, we found the system present in vast majority in *B. anthracis*. Given the relatively recent evolution of *B. anthracis* from a parental *B. cereus* subgroup, it would be reasonable to conjecture that the presence of Nhi in *B. anthracis,* and particularly on the pXO1 plasmid, originates from this common parental source and was stabilised within members of the species due to their clonal nature.

Septu, AbiO, Nhi and DarTG systems are, in general, over-represented compared to their phylum, as their abundance in the *B. cereus* group exceeds by 8–13% their abundance in Bacillota. On average, CRISPR-Cas is less prevalent in the group than expected compared to phylum Bacillota. However, this system is close to absent in *B. anthracis*, with 0.3% of assemblies containing a CRISPR-Cas system, while 97.7% of* B. cytotoxicus* genomic assemblies encode at least one such system. This relative rarity of CRISPR-Cas in the *B. cereus* group has previously been reported, revealing that many genomes in this group are devoid of, or harbour defective, CRISPR-Cas systems^7^. In the group, CRISPR-Cas systems seem to act as barriers to HGT, encouraging the evolution of *B. cereus* population towards inactivation of CRISPR-Cas systems in favour of MGEs acquisition and genetic remodelling. In addition, in our analyses there was a slight negative association between CRISPR-Cas and RM systems. Knowing the heterogeneity of CRISPR-Cas distribution across the *B. cereus* species, its overall low abundance in the group compared to other prokaryotes and the tendency of this system to be degenerated in the *B. cereus* group, we hypothesise that RM and CRISPR-Cas may be less compatible defence strategies than in other bacterial groups.

As previously mentioned, defence systems are often encoded together within DIs and transferred between genomes by MGEs^1,2^. As a result, a group of systems could be transferred together by one single transfer event. Evidence also shows that some defence systems can work in association to increase the antiviral resistance of bacteria, *i.e.* type II CRISPR-Cas and type II R-M, Zorya II with Druantia II and ietAS, and more^42,43^. Our analysis indicated that DarTG, AbiO, CRISPR-Cas, DRT, AbiQ and AVAST have a tendency to be encoded together within genomes of the *B. cereus* group. A recent study by Wu et al*.* (2024)^43^ has highlighted synergies between defence systems in *E*. *coli*, also revealing that negative association of systems within genomes may not be a consequence of mechanistic incompatibility. Rather, systems’ genomic co-occurrence seems to mirror the variability of the bacterial arsenal, with substantial intraspecific differences, and cooperation between systems that may be linked to target phage specificity^43^.

Overall, the *B. cereus* group seems to rely on a rich, diverse and possibly multi-layered arsenal of strategies to handle phages’ attacks. On the one hand, RM systems seem to be the main defence allowing cell survival, in particular RM type I and II(G). On the other hand, genomic assemblies have a wide array of cell suicide alternatives, among which some are extremely abundant (*i.e.* Septu, Lamassu and Gabija).

### The *B. cereus* mobile defensome

Antiviral defence systems seem to act as a community resource, shared between closely related strains via MGEs. Knowing this high mobility of defence systems and their prevalence in MGEs, we expected to find many *B. cereus* group’s defence systems encoded into plasmids. Although many validated systems are indeed encoded in plasmids, many can also be found in the chromosome of complete genomes from members of the *B. cereus* group. Still, some tendencies have been identified, for instance validated RM systems encoded mostly on the chromosome while validated Nhi, AbiO and most AbiQ systems were found on plasmids. In fact, RM_II systems have been described as selfish units able to stabilise themselves into a bacterial genome by degrading the cell chromosome in the event of gene loss^44^. In other studies, Nhi and lactococcal abi systems have indeed been described as systems mostly found into plasmids^35,43,45^. The fact that many systems can be found on both the chromosome and plasmids attests of the tendency of defence systems to mobility and does not exclude the possibility of some of these defence systems being encoded by other forms of MGEs inserted into the chromosome, such as transposons, prophages, conjugative elements, and more, as this group is characterised by its high diversity of extrachromosomal genetic material, including but not limited to plasmids.

## Conclusion

Our findings provide a picture of a rich and complex antiviral arsenal in the *B. cereus* group and highlight the heterogeneity of defence systems’ distribution in this genetically close population of bacteria. Future studies involving in vivo experiments may bridge the gap in our understanding of how these defence systems may impact phage-host interactions, and possibly, interactions with other MGEs.

## Supplementary Information

Supplementary Legends.

Supplementary Data 1.

Supplementary Data 2.

Supplementary Data 3.

Supplementary Data 4.

Supplementary Data 5.

Supplementary Data 6.

Supplementary Data 7.

Supplementary Data 8.

Supplementary Information.

## Data Availability

All data generated or analysed during this study are included in this published article and its supplementary information files. Referenced datasets from DefenseFinder Webservice analysed in this study can be found at: https://defensefinder.mdmlab.fr/.
