# Supplementary material for: Antiviral defence arsenal across members of the Bacillus cereus group
Source: Sci Rep. 2025 Feb 10;15:4958. doi: 10.1038/s41598-025-86748-8 (PMC11811056; doi:10.1038/s41598-025-86748-8)
Supplement: Supplementary file 1 — Supplementary Legends. [file 41598_2025_86748_MOESM1_ESM.pdf]

Description of Additional Supplementary Data for

Antiviral defence arsenal across members of the *Bacillus cereus* group

Elise July and Annika Gillis*

*Corresponding author. E-mail address: annika.gillis@uclouvain.be

Supplementary Data 1.xls – List of GenBank and RefSeq accession numbers used in this work (NCBI database).

This table contains the list of the 6366 genomic assemblies analysed in this work, retrieved from the NCBI database in March 2024.

Supplementary Data 2.xls – All defence systems predicted by DefenseFinder in the *Bacillus cereus* group.

This table contains all the defence systems detected by DefenseFinder with default parameters, plus a novel system type and subtype attribution for each system, for data harmonisation.

Supplementary Data 3.xls – All defence systems predicted by PADLOC in the *Bacillus cereus* group.

This table contains all the defence systems detected by PADLOC with default parameters, plus a novel system type and subtype attribution for each system, for data harmonisation.

Supplementary Data 4.xls – Table of correspondence of defence systems detected by DefenseFinder and PADLOC.

This table is built as a comparison between the lists of different systems detected by DefenseFinder and PADLOC. A novel system type and subtype was attributed to each DefenseFinder and PADLOC corresponding systems pairs.

Supplementary Data 5.xls – Table of identical defence systems predicted as different by DefenseFinder and PADLOC.

This table comprises the 509 systems that were composed of identical sets of proteins between DefenseFinder and PADLOC. A unique system type has been found for most of these, but no consensus was found for a few PsyrTA/ShosTA, Pycsar/CBASS_I, Stk2/PD-T4-6 and AbiO/Nhi systems pairs.

Supplementary Data 6.xls – Table of similar predicted defence systems by either DefenseFinder or PADLOC.

This table comprises a total of 2505 different systems which have at least one protein in common detected by DefenseFinder and PADLOC.

Supplementary Data 7.xls – Table of validated defence systems in the *Bacillus cereus* group.

This table comprises the 16,584 validated defence systems, that were predicted by both DefenseFinder and PADLOC.

Supplementary Data 8.xls – Global table of different defence systems predicted by DefenseFinder and/or PADLOC.

This table comprises the 83,738 systems that were predicted by DefenseFinder and/or PADLOC in the *Bacillus cereus* group. This dataset comprises the systems analysed in this work, cleaned up of duplicate systems (see Methods). In this table, 486 systems labelled as DefenseFinder systems (score of 1) were in fact a match to a PDC-x duplicate partner (see Methods). Only 16,098 systems out of 16,854 are thus scored as 0 in this table.
