# Supplementary material for: Antiviral defence arsenal across members of the Bacillus cereus group
Source: Sci Rep. 2025 Feb 10;15:4958. doi: 10.1038/s41598-025-86748-8 (PMC11811056; doi:10.1038/s41598-025-86748-8)
Supplement: Supplementary file 10 — Supplementary Information. [file 41598_2025_86748_MOESM10_ESM.docx]

Supplementary Information for

**Antiviral defence arsenal across members of the *Bacillus cereus* group**

Elise July and Annika Gillis^*^

*Corresponding author. E-mail address: [annika.gillis@uclouvain.be](mailto:annika.gillis@uclouvain.be)

This file includes:

**Supplementary figures**

**Supplementary Fig. S1. Representation of defence systems identified in complete genomes of the *Bacillus cereus* group.**

**Supplementary Fig. S2. Distribution of defence systems and genes in complete genomes of the *Bacillus cereus* group.**

**Supplementary Fig. S3. Comparison of abundance of defence systems (% genome) between *Bacillus cereus* *sensu lato* and Bacillota.**

**Supplementary Fig. S4. Representation of defence strategies in the *Bacillus cereus* group.**

**Supplementary Fig. S5. Overview of all defence systems** **predicted per** **species in the *Bacillus cereus* group.**

**Supplementary Fig. S6. Total number of defence systems found in the *Bacillus cereus* group pan-genome.**

**Supplementary Fig. S7. Distribution of validated defence systems and genes in the *Bacillus cereus* group.**

**Supplementary Fig. S8. Representation of defence strategies for validated systems in the *Bacillus cereus* group.**

**Supplementary Fig. S9. Comparison of abundance of validated systems (% genome) between *Bacillus cereus sensu lato* and Bacillota.**

**Supplementary Fig. S10. Representation of relative distance between validated defence systems.**

**Supplementary tables**

**Supplementary Table S1. Summary of all defence systems detected in the *Bacillus cereus* group.**

**Supplementary Table S2. Summary of all CRISPR-Cas systems validated in the *Bacillus cereus* group.** Prevalence calculated as the sum of assemblies^(*1)^ / Total count of assemblies^(*2)^ *100.

**Part A. Supplementary figures for complete dataset**


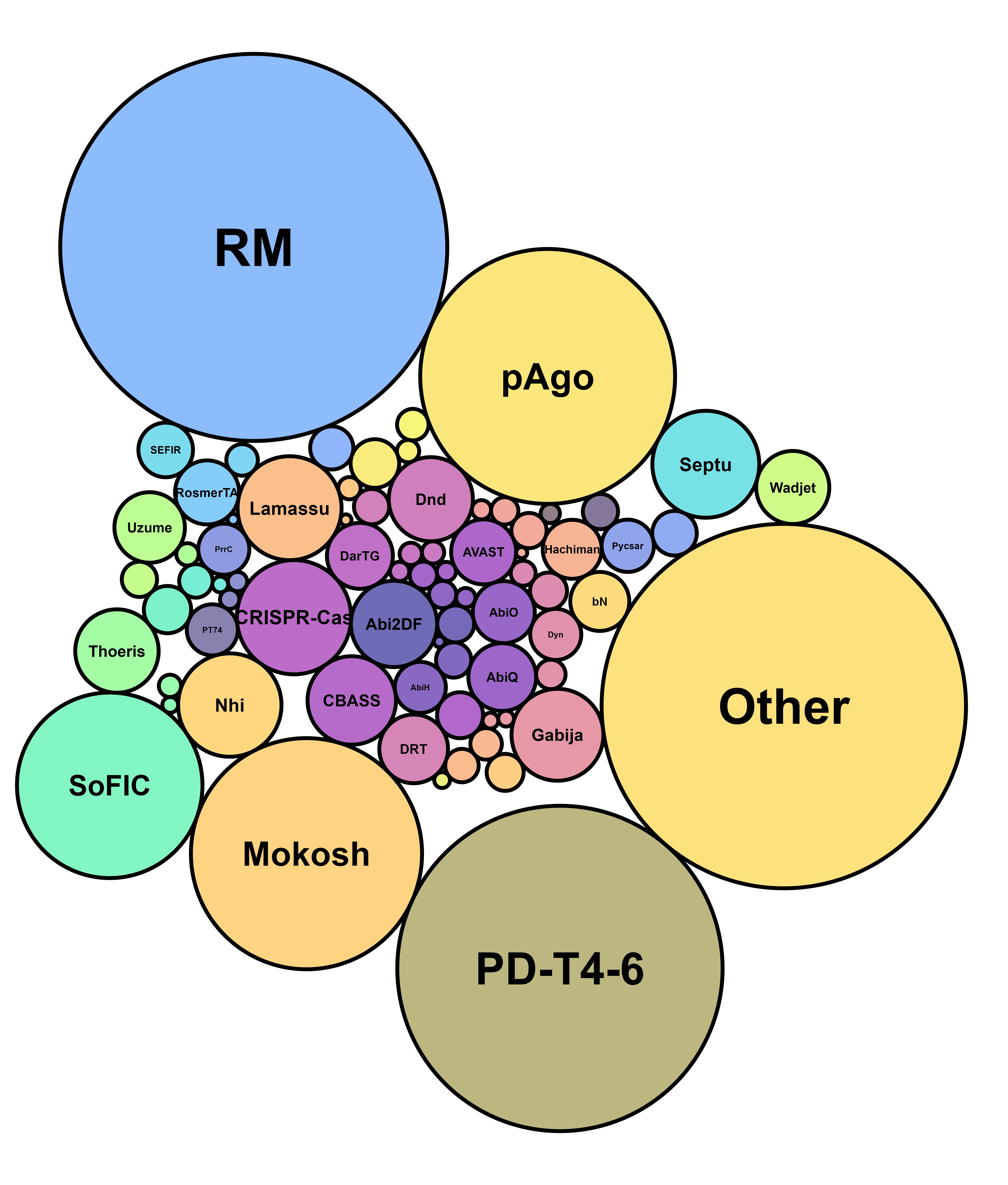


**Supplementary Fig. S1. Representation of defence systems identified in complete genomes of the *Bacillus cereus* group.** A total of 5807 defence systems were encoded in complete genomes (n = 460). The most abundant systems are RM, PDC-x (other), PD-T4-6, pAgo and Mokosh. Defence systems for which less than 20 occurrences in the *B. cereus* group have been counted are not labelled. Size of the circle represents the total number of predicted defence systems. Abbreviations: Dyn (Dynamins), bN (NRL_bNACHT), PT74 (PD-T7-4).

| **a**  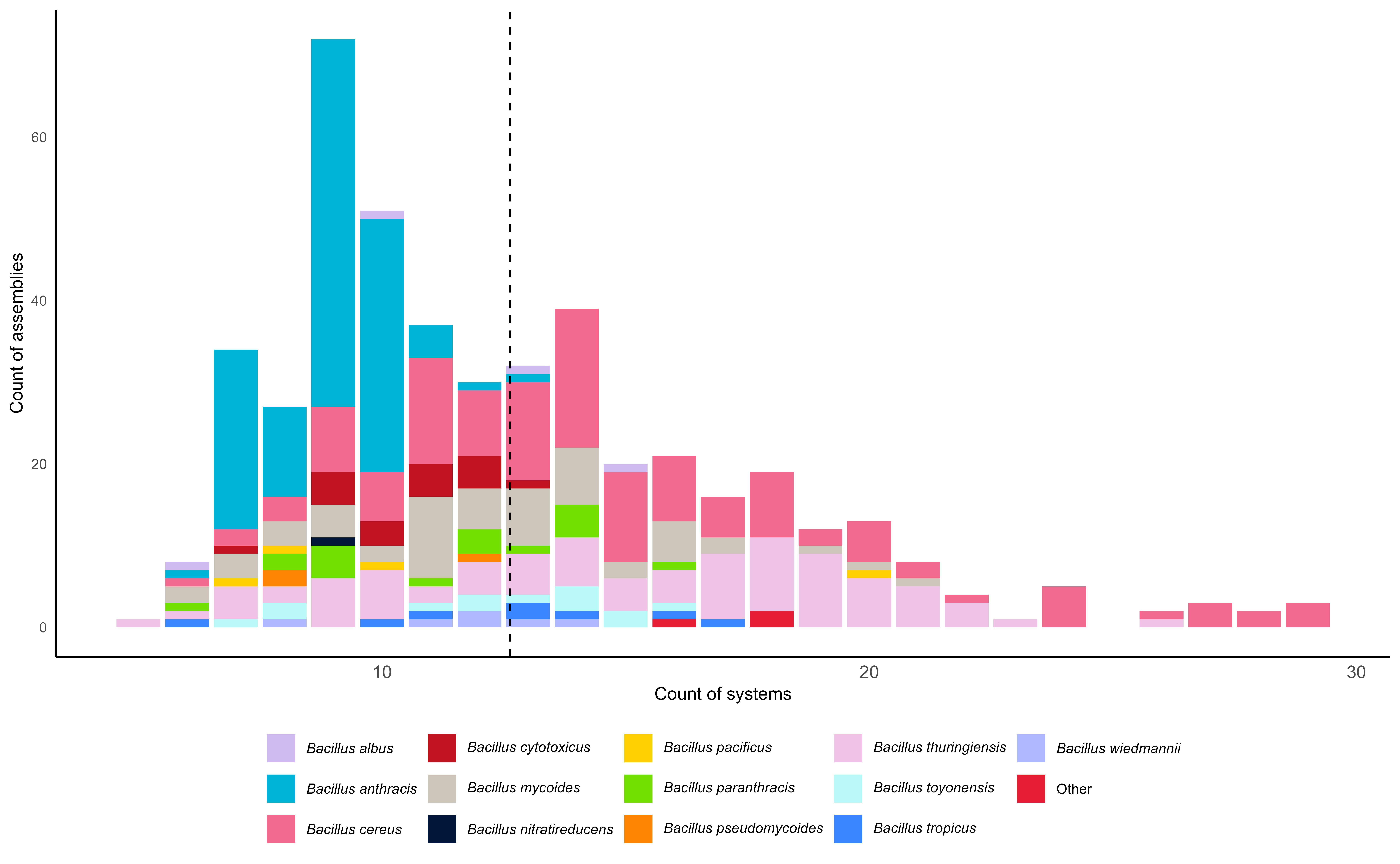 |
| --- |
| **b**  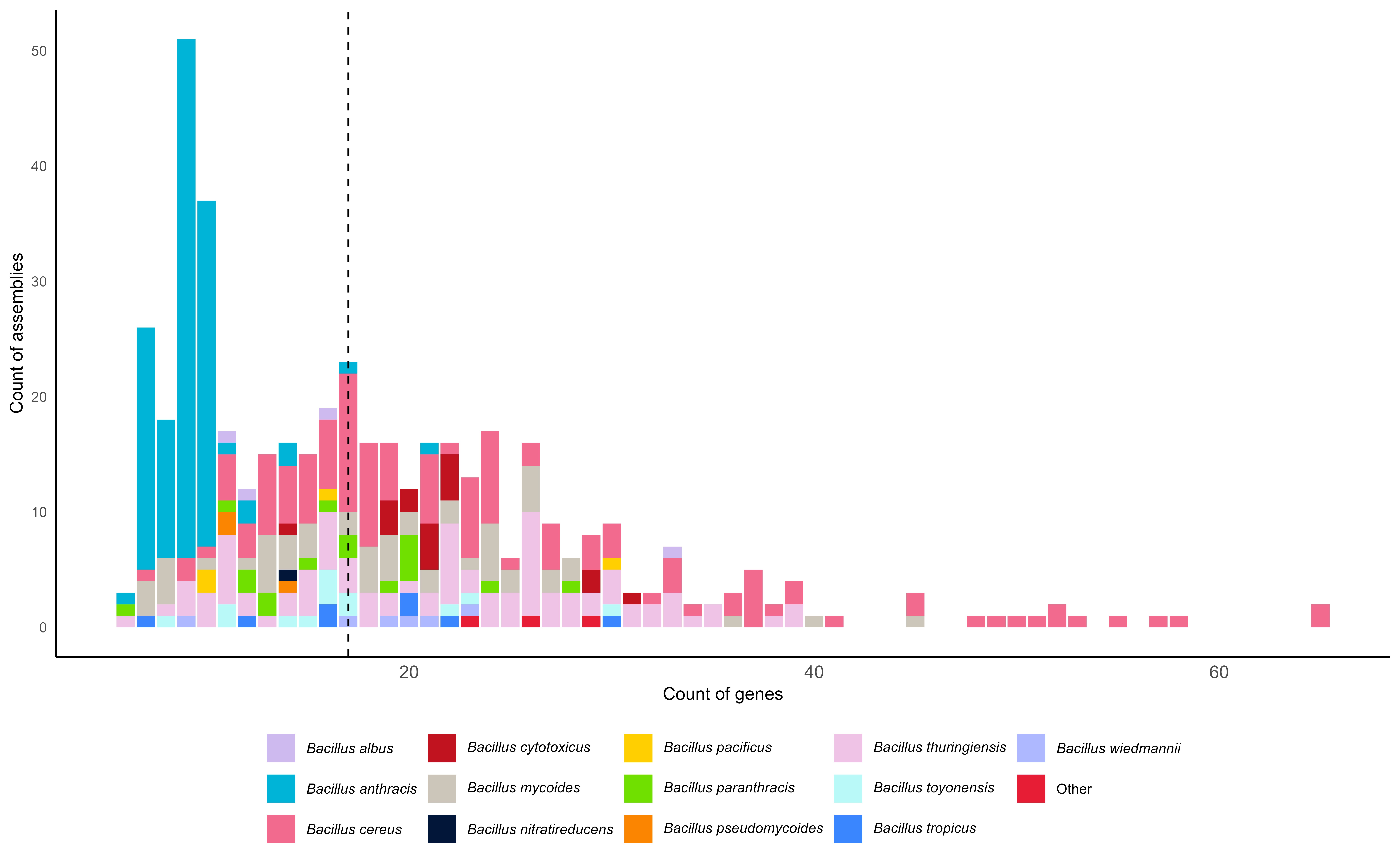 |
| **c**  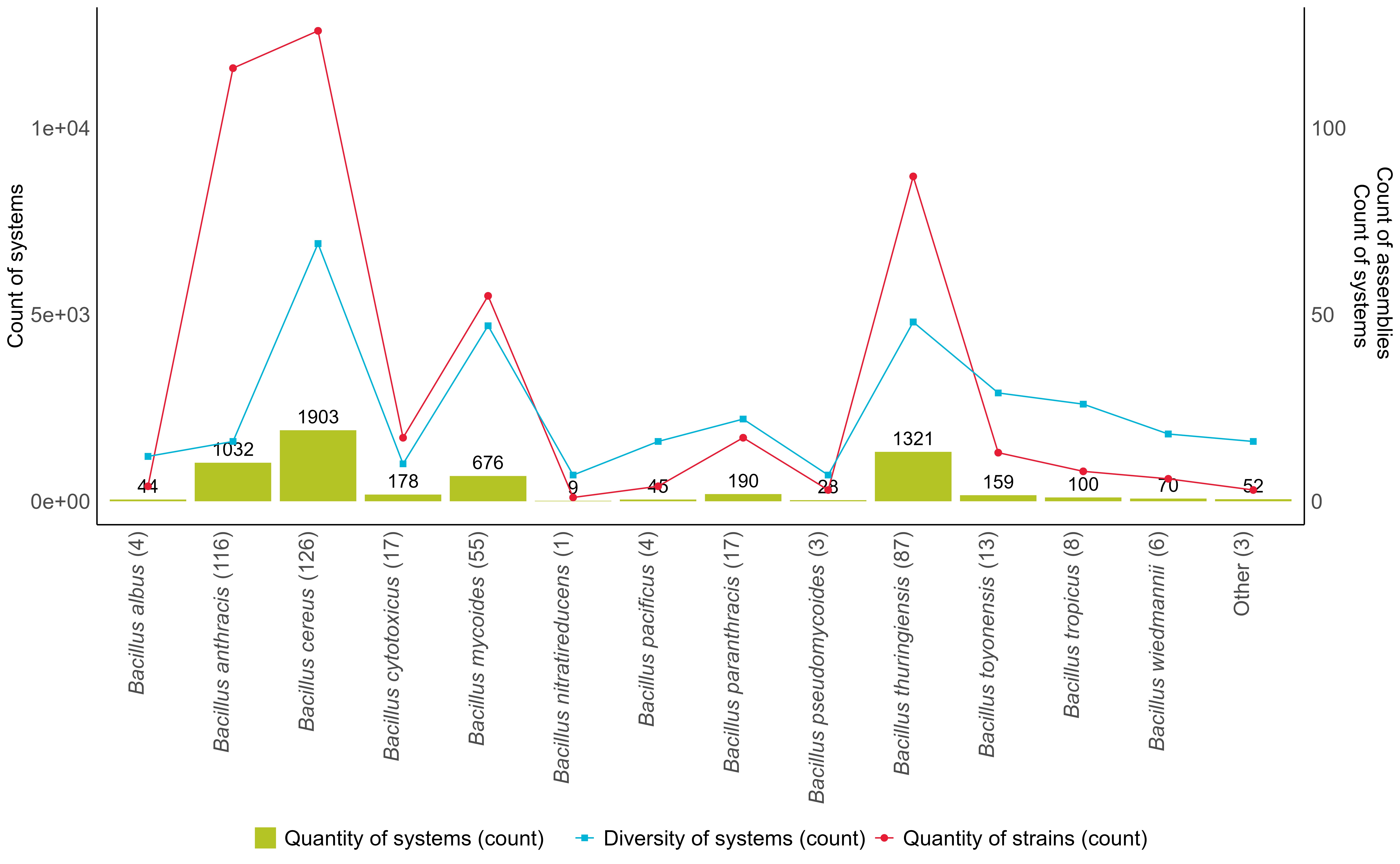 |

**Supplementary Fig. S2. Distribution of defence systems and genes in complete genomes of the *Bacillus cereus* group. (a)** Distribution of the total number of defence systems per genomic assembly (min = 5, max = 29, mean = 12.6, median = 11.5). Dashed line, average across the group. (**b)** Distribution of the total number of defence genes per genomic assembly (min = 6, max = 65, mean = 18.5, median = 17). Dashed line, average across the group**. (c)** Abundance of systems per species in the *B. cereus* group. Species with the most genomic assemblies cumulate higher quantity and diversity of systems. Overall, *B. cereus sensu stricto* (*s.s.*) and *Bacillus thuringiensis* gather the highest number of systems for this bacterial group. Red line, count of assemblies. Blue line, count of different types of defence systems.


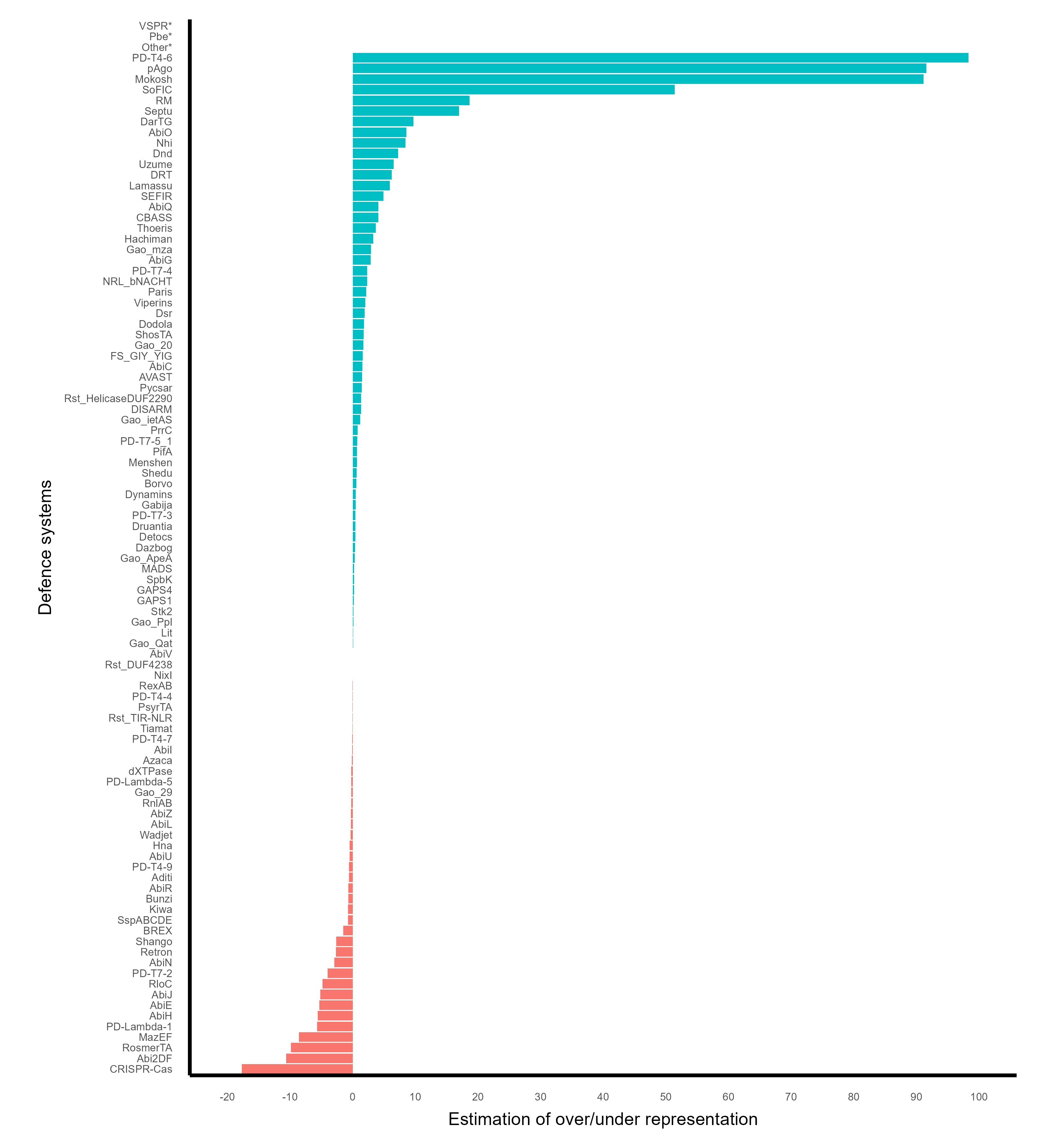


**Supplementary Fig. S3. Comparison of abundance of defence systems (% genome) between *Bacillus cereus* *sensu lato* and Bacillota.** The estimation of under or over representation of defence systems encoded in the *B. cereus* group compared to systems encoded in phylum Bacillota was calculated as the difference between the abundance in the *B. cereus* group genomic assemblies and the abundance in Bacillota complete genomes, as provided by DefenseFinder Webservice (see Methods, Equation 1). The * indicates that the abundance per genomes in Bacillota is not available. For defence system “Dynamins” prevalence is approximated by “Eleos” prevalence, and for defence system “Abi2DF” by “AbiD” prevalence.


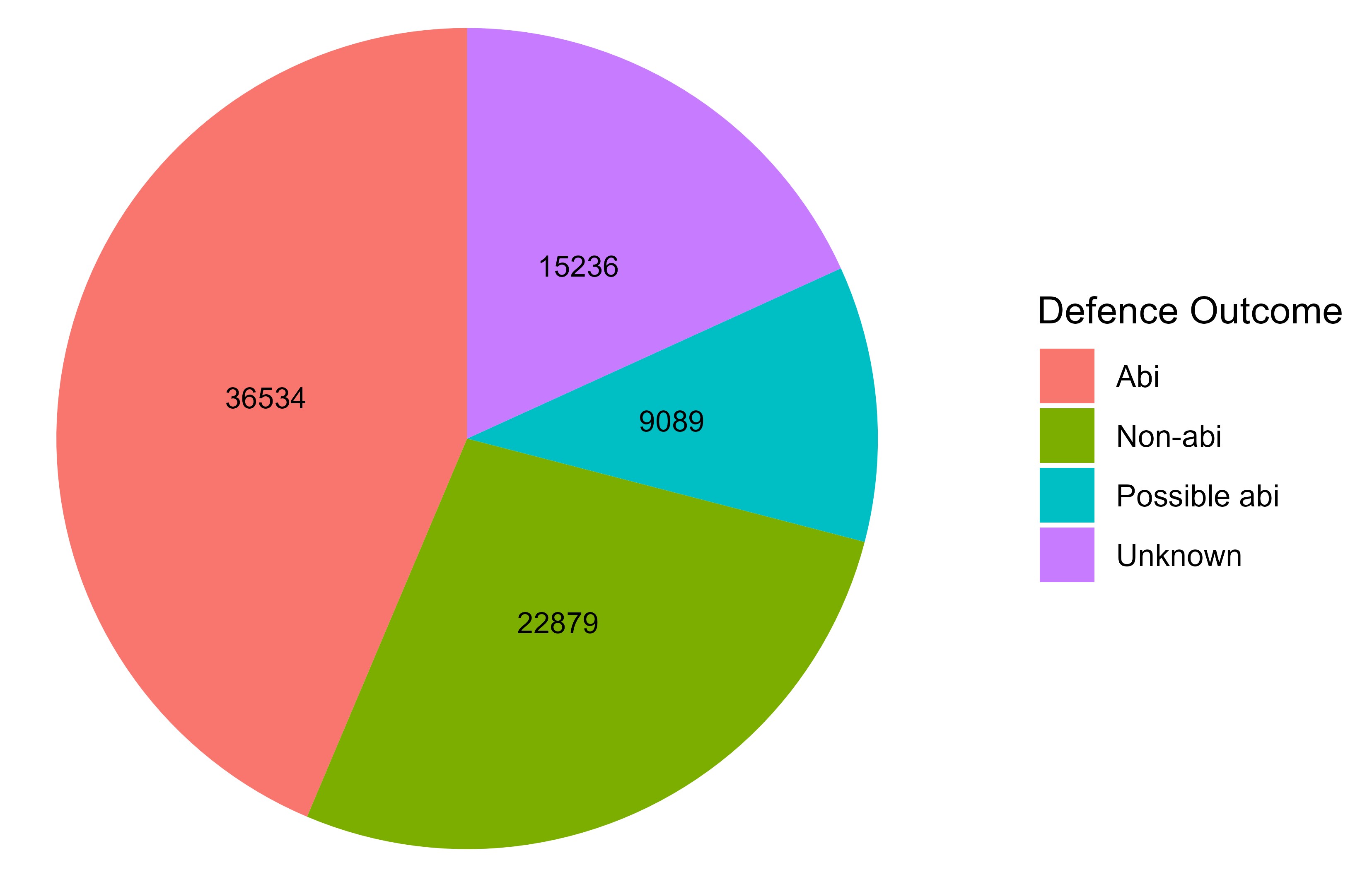


**Supplementary Fig. S4. Representation of defence strategies in the *Bacillus cereus* group.** A vast majority of defence systems display abi outcomes. All but two genomic assemblies possess at least an abi system, and all but 10 assemblies possess at least one non-abi system. Defence systems cell outcomes indicated in colour: red, abi; green, non-abi; cyan, possible abi; mauve, unknown.


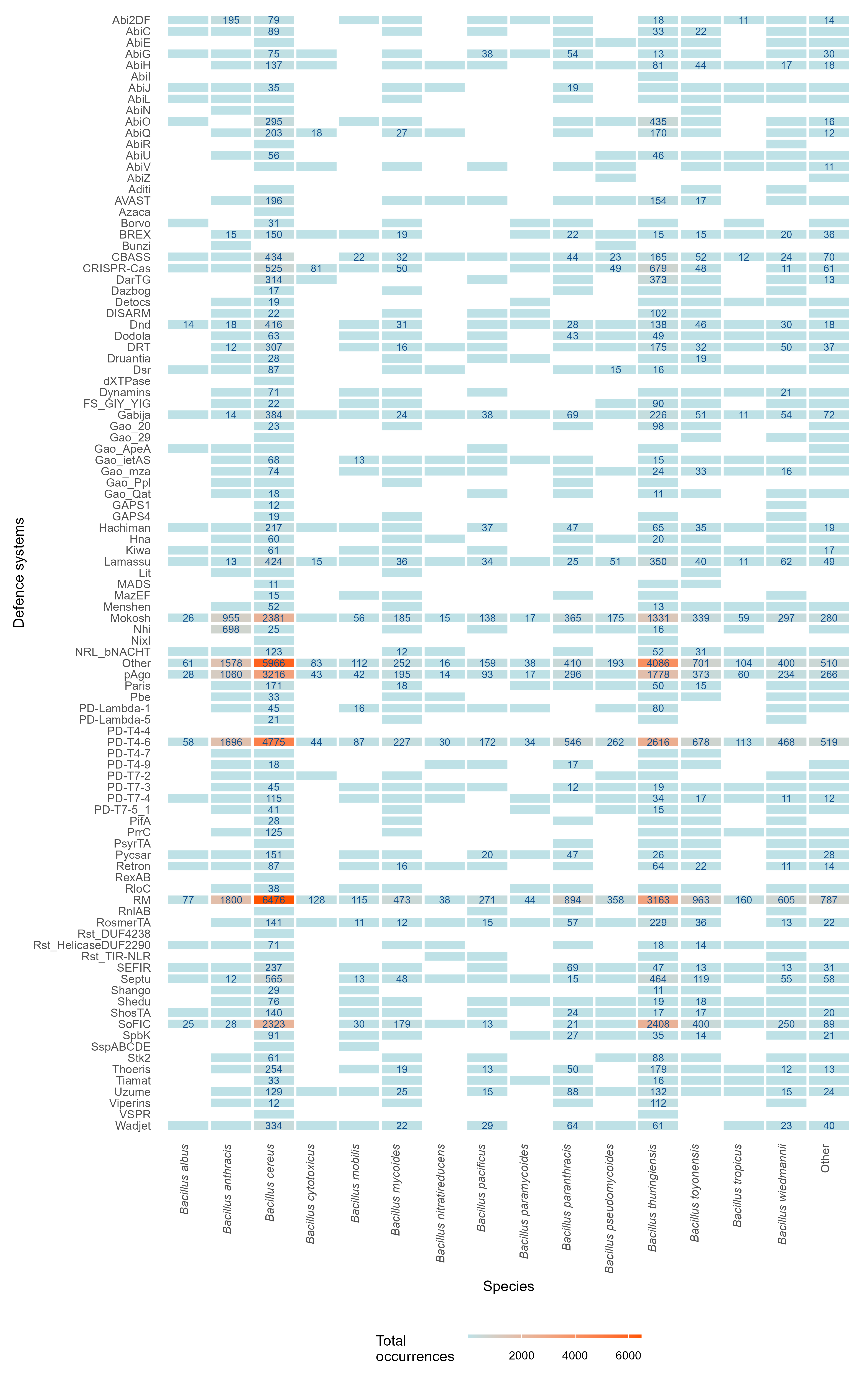


**Supplementary Fig. S5. Overview of all defence systems** **predicted per** **species in the *Bacillus cereus* group.** Except for Nhi and rare systems (< 10 occurrences), the most abundant species, *B. cereus sensu stricto* and *Bacillus* *thuringiensis* possess the most number and diverse systems. The colour scale represents the total count of defence systems. These counts are displayed in boxes, shown only for > 10 occurrences.

**Part B. Supplementary figures for validated defence systems**

“Validated systems” are identical systems detected by both PADLOC and DefenseFinder, possessing the same system type, number and identity of protein, within a single assembly (see Methods).


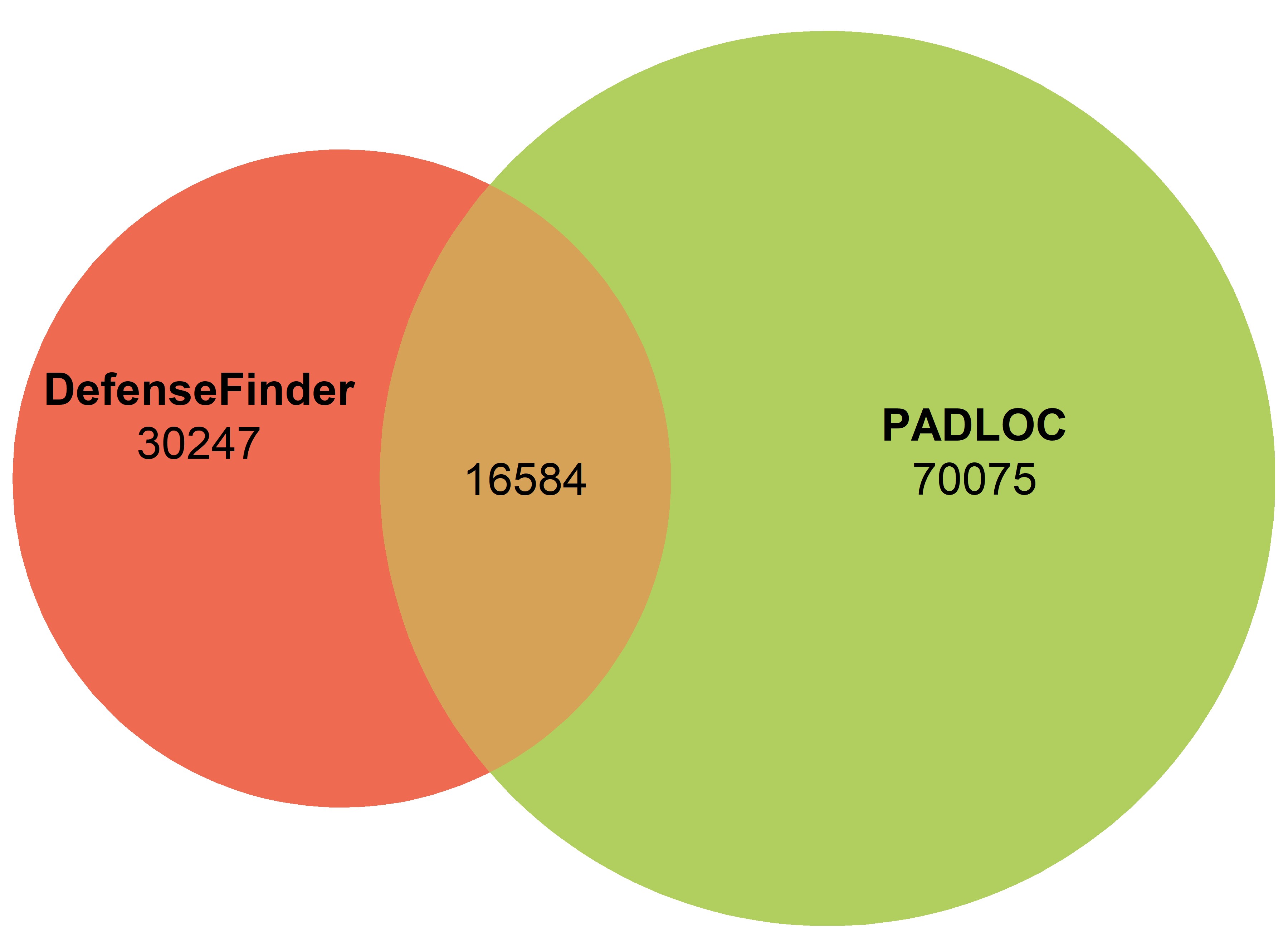


**Supplementary Fig. S6. Total number of defence systems found in the *Bacillus cereus* group pan-genome.** Out of the 100,322 defence systems predicted in raw dataset, 16,584 systems were duplicates identified by both DefenseFinder and PADLOC and considered as validated (confirmed) systems. Overall, a total of 83,738 different systems are predicted in the *B. cereus* group pan-genome.

**a**

| **b**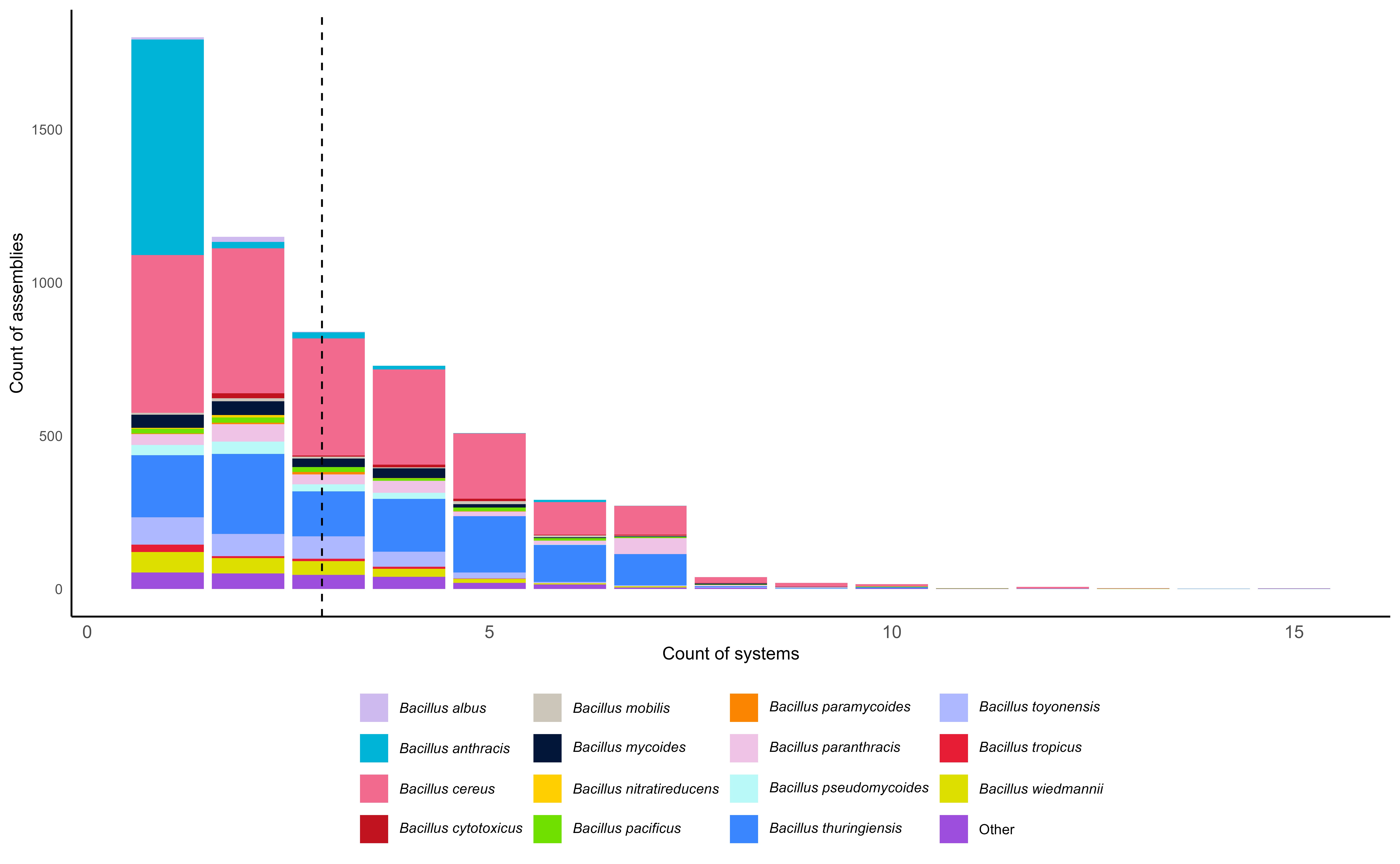 |
| --- |
| 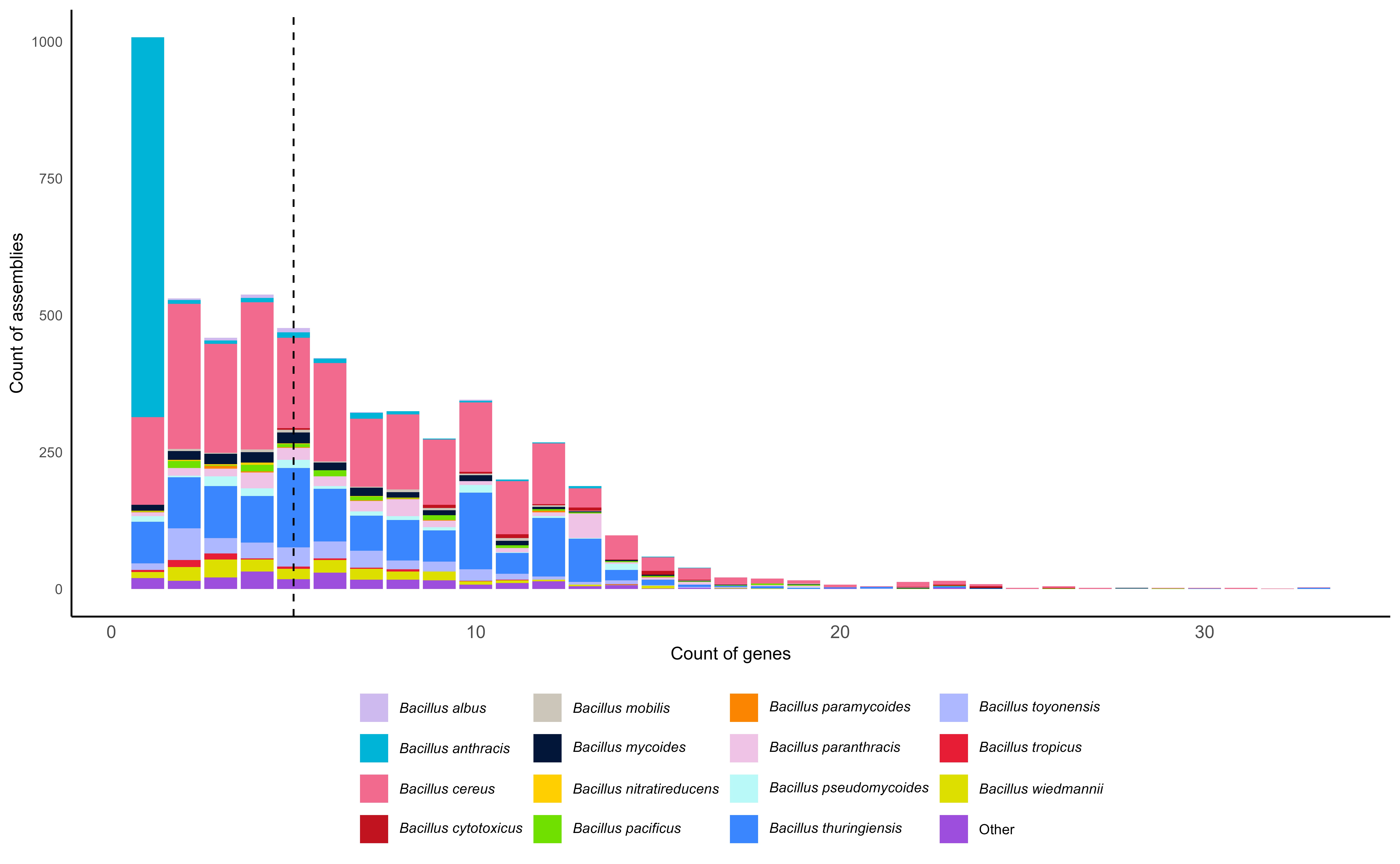 |
| **c**  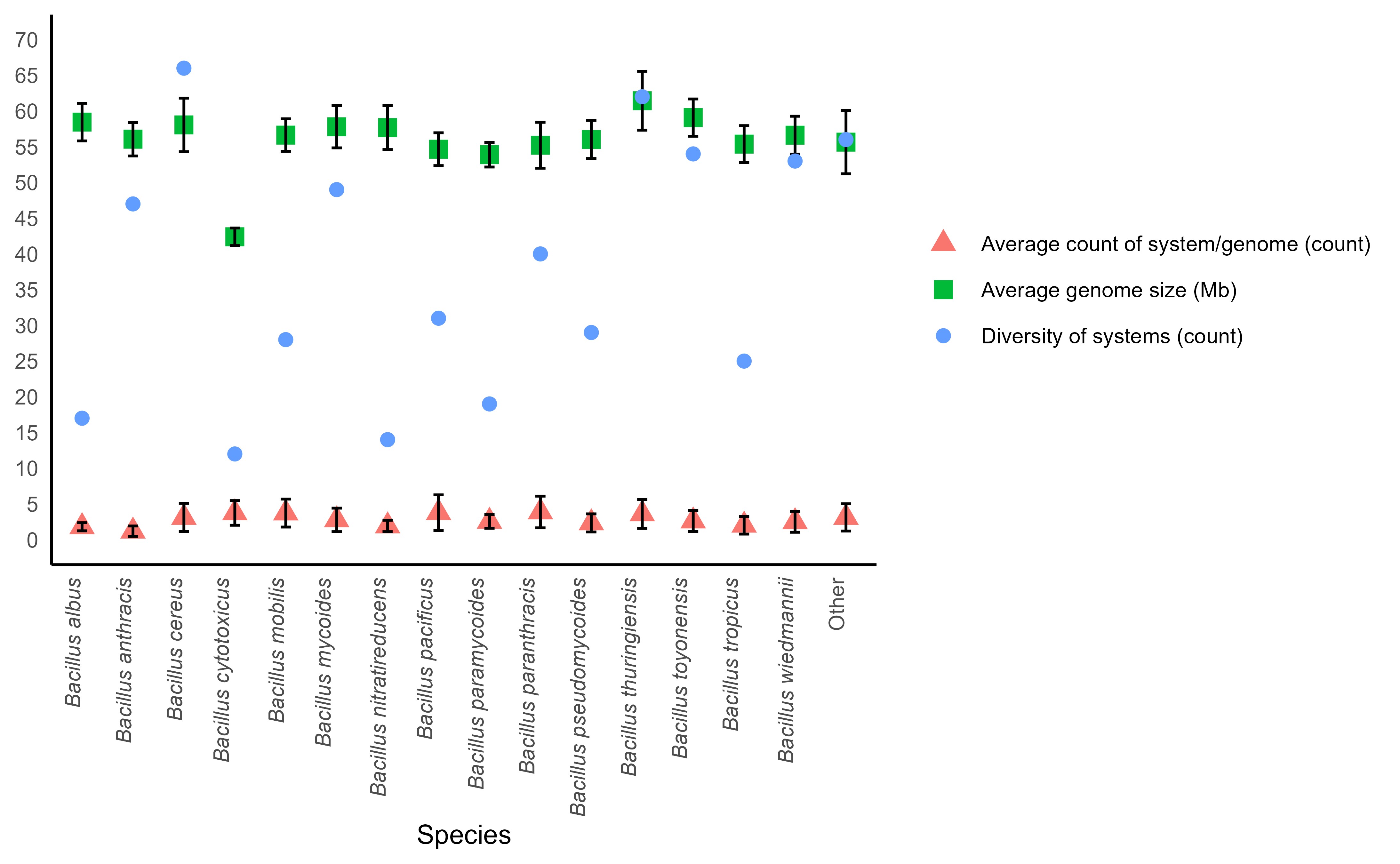 |

**Supplementary Fig. S7. Distribution of validated defence systems and genes in the *Bacillus cereus* group. (a)** Distribution of the total number of defence systems per genomic assembly (min = 1, max = 15, mean = 3, median = 2). Dashed line; average across the group. (**b)** Distribution of the total number of defence genes per genomic assembly (min = 1, max = 33, mean = 6, median = 5). Dashed line; average across the group**. (c)** Average number of defence systems and genomic size per strain across species in the *B. cereus* group. For average genomic assembly size (in Mb) Y-axis should be divided by 10. Standard deviation calculated based on the number of systems per genomic assemblies, for each species.


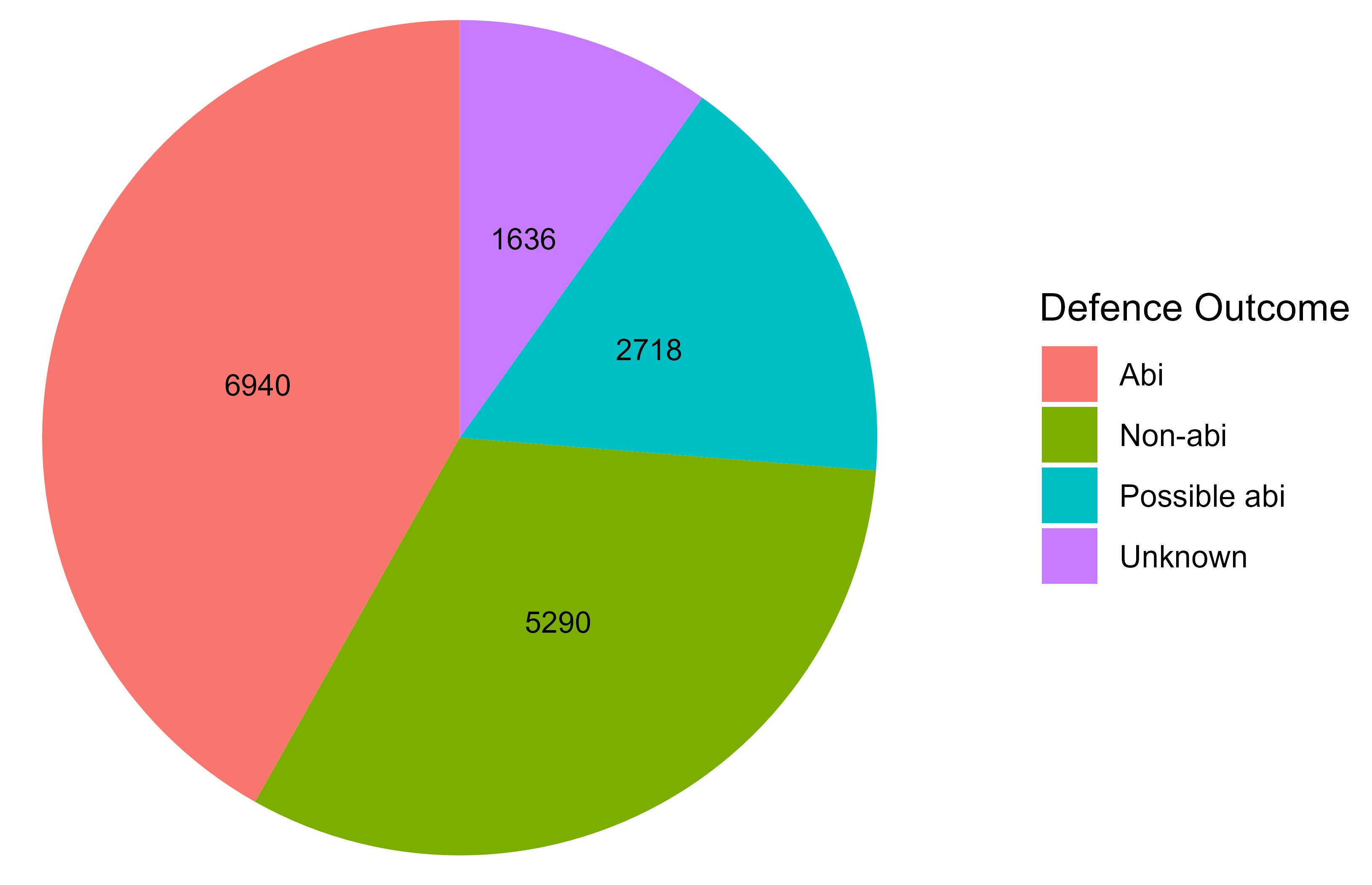


**Supplementary Fig. S8. Representation of defence strategies for validated systems in the *Bacillus cereus* group.** A vast majority of defence systems display abi strategies. Defence outcome indicated in colour: red, abi; green, non-abi; cyan, possible abi; mauve, unknown.


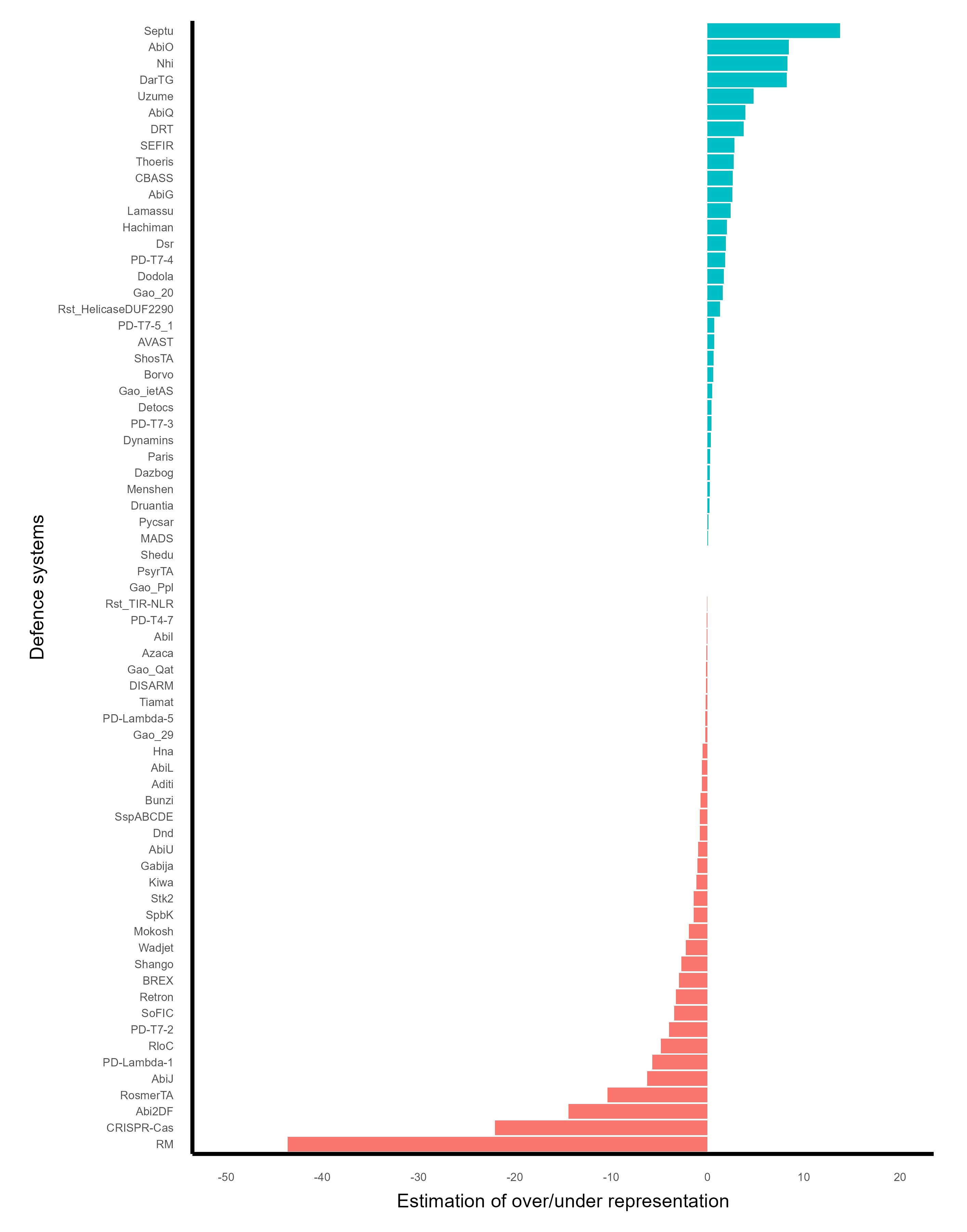


**Supplementary Fig. S9. Comparison of abundance of validated systems (% genome) between *Bacillus cereus sensu lato* and Bacillota.** The estimation of under or over representation systems encoded in the *B. cereus* group compared to defence systems encoded in phylum Bacillota is calculated as the difference between the abundance in the *B. cereus* group assemblies and the abundance in Bacillota complete genomes, as provided by DefenseFinder Webservice (see Methods, Equation 1). For defence system “Dynamins” prevalence is approximated by “Eleos” prevalence, and for defence system “Abi2DF” by “AbiD” prevalence.


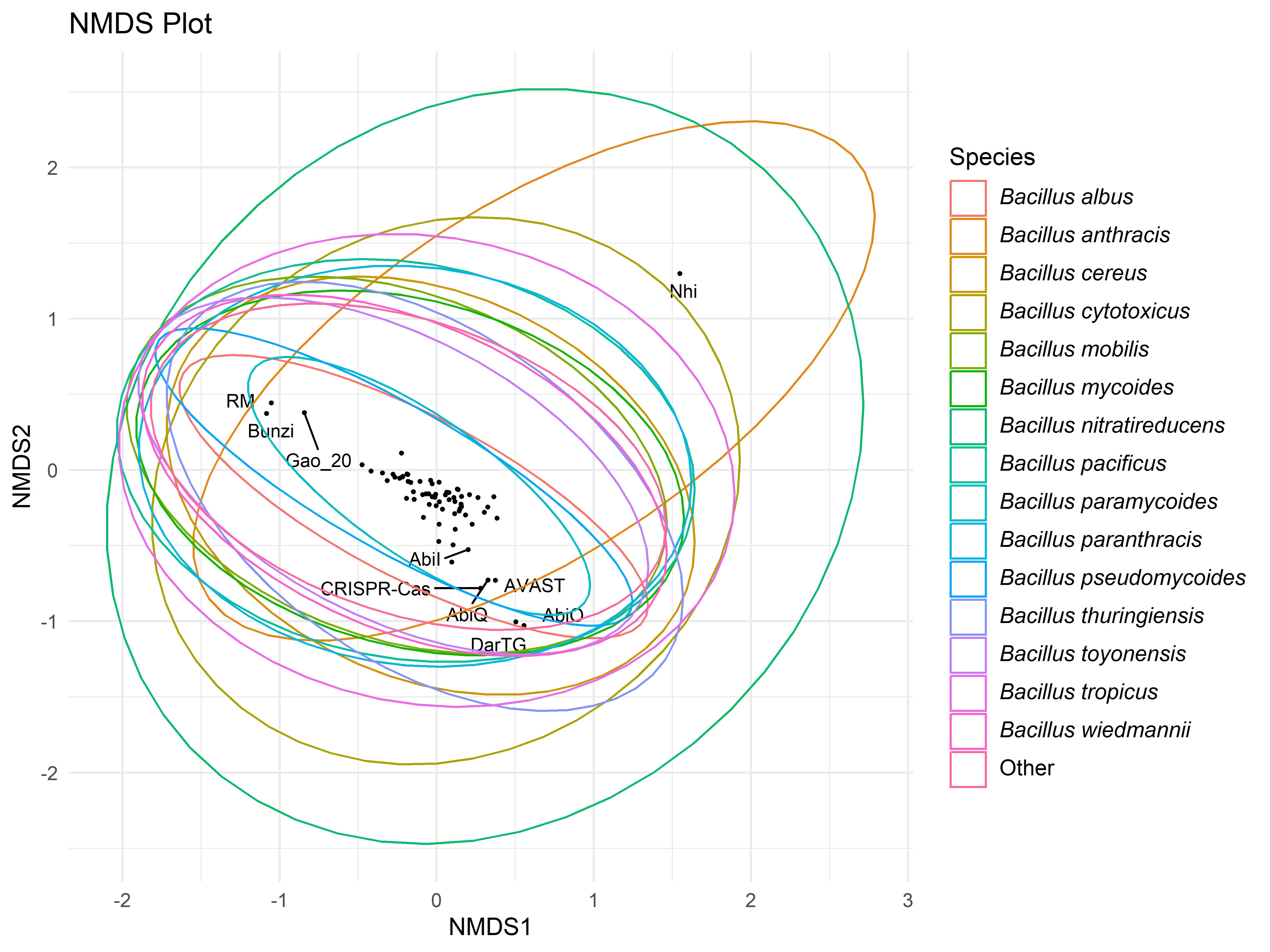


**Supplementary Fig. S10. Representation of relative distance between validated defence systems.** The information of system’s presence-absence within genomic assemblies was transformed into a Bray-Curtis distance matrix, where distance represents the pairwise difference between genomes as coordinates on a map. The Non-metric MultiDimensional Scaling (NMDS) analysis is used to generate scores that interpret the distances and group the genomes based on their closeness.

**Part C. Supplementary tables**

**Supplementary Table S1. Summary of all defence systems detected in the *Bacillus cereus* group.**

| **System** | **Count per ass.** | **Total** | **Grand total** | **System** | **Count per ass.** | **Total** | **Grand total** | **System** | **Count per ass.** | **Total** | **Grand total** | **System** | **Count per ass.** | **Total** | **Grand total** |
| --- | --- | --- | --- | --- | --- | --- | --- | --- | --- | --- | --- | --- | --- | --- | --- |
| Abi2DF | 1 | 340 | 343 | Dodola | 1 | 182 | 189 | NRL_bNACHT | 1 | 237 | 240 | RM | 1 | 400 | 6339 |
|  | 2 | 3 |  |  | 2 | 5 |  |  | 2 | 3 |  |  | 2 | 3295 |  |
| AbiC | 1 | 167 | 169 |  | 4 | 2 |  | Other | 1 | 1427 | 5622 |  | 3 | 1683 |  |
|  | 2 | 2 |  | DRT | 1 | 580 | 616 |  | 2 | 1907 |  |  | 4 | 630 |  |
| AbiE | 1 | 22 | 22 |  | 2 | 33 |  |  | 3 | 916 |  |  | 5 | 243 |  |
| AbiG | 1 | 213 | 219 |  | 3 | 3 |  |  | 4 | 545 |  |  | 6 | 53 |  |
|  | 2 | 6 |  | Druantia | 1 | 57 | 63 |  | 5 | 592 |  |  | 7 | 22 |  |
| AbiH | 1 | 311 | 316 |  | 2 | 6 |  |  | 6 | 128 |  |  | 8 | 11 |  |
|  | 2 | 5 |  | Dsr | 1 | 144 | 149 |  | 7 | 90 |  |  | 9 | 2 |  |
| AbiI | 1 | 2 | 2 |  | 2 | 5 |  |  | 8 | 12 |  | RnlAB | 1 | 17 | 17 |
| AbiJ | 1 | 78 | 81 | DUF4238 | 1 | 1 | 1 |  | 9 | 4 |  | RosmerTA | 1 | 548 | 551 |
|  | 2 | 3 |  | dXTPase | 1 | 1 | 1 |  | 10 | 1 |  |  | 2 | 3 |  |
| AbiL | 1 | 36 | 36 | Dynamins | 1 | 104 | 108 | pAgo | 1 | 4044 | 5851 | Rst_HelicaseDUF2290 | 1 | 116 | 118 |
| AbiN | 1 | 3 | 3 |  | 2 | 4 |  |  | 2 | 1757 |  |  | 2 | 2 |  |
| AbiO | 1 | 426 | 595 | FS_GIY_YIG | 1 | 127 | 127 |  | 3 | 43 |  | Rst_TIR-NLR | 1 | 10 | 10 |
|  | 2 | 168 |  | Gabija | 1 | 811 | 885 |  | 4 | 6 |  | SEFIR | 1 | 424 | 428 |
|  | 3 | 1 |  |  | 2 | 65 |  |  | 5 | 1 |  |  | 2 | 4 |  |
| AbiQ | 1 | 426 | 435 |  | 3 | 7 |  | Paris | 1 | 244 | 258 | Septu | 1 | 995 | 1170 |
|  | 2 | 9 |  |  | 4 | 2 |  |  | 2 | 11 |  |  | 2 | 152 |  |
| AbiR | 1 | 9 | 9 | Gao_20 | 1 | 128 | 128 |  | 3 | 3 |  |  | 3 | 18 |  |
| AbiU | 1 | 121 | 124 | Gao_29 | 1 | 18 | 18 | Pbe | 1 | 54 | 54 |  | 4 | 4 |  |
|  | 2 | 3 |  | Gao_ApeA | 1 | 27 | 27 | PD-Lambda-1 | 1 | 170 | 174 |  | 5 | 1 |  |
| AbiV | 1 | 48 | 48 | Gao_ietAS | 1 | 128 | 134 |  | 2 | 4 |  | Shango | 1 | 54 | 56 |
| AbiZ | 1 | 1 | 2 |  | 2 | 6 |  | PD-Lambda-5 | 1 | 26 | 26 |  | 2 | 2 |  |
|  | 2 | 1 |  | Gao_mza | 1 | 185 | 186 | PD-T4-4 | 1 | 1 | 1 | Shedu | 1 | 132 | 132 |
| Aditi | 1 | 6 | 6 |  | 2 | 1 |  | PD-T4-6 | 1 | 495 | 6334 | ShosTA | 1 | 152 | 201 |
| AVAST | 1 | 380 | 384 | Gao_Ppl | 1 | 15 | 15 |  | 2 | 5688 |  |  | 2 | 49 |  |
|  | 2 | 4 |  | Gao_Qat | 1 | 41 | 42 |  | 3 | 150 |  | SoFIC | 1 | 2095 | 3497 |
| Azaca | 1 | 1 | 1 |  | 2 | 1 |  |  | 4 | 1 |  |  | 2 | 638 |  |
| Borvo | 1 | 48 | 48 | GAPS1 | 1 | 13 | 13 | PD-T4-7 | 1 | 11 | 11 |  | 3 | 650 |  |
| BREX | 1 | 163 | 237 | GAPS4 | 1 | 26 | 26 | PD-T4-9 | 1 | 56 | 56 |  | 4 | 106 |  |
|  | 2 | 74 | 2 | Hachiman | 1 | 388 | 416 | PD-T7-2 | 1 | 21 | 21 |  | 5 | 7 |  |
| Bunzi | 1 | 2 |  |  | 2 | 25 |  | PD-T7-3 | 1 | 110 | 111 |  | 6 | 1 |  |
| CBASS | 1 | 492 | 677 |  | 3 | 3 |  |  | 2 | 1 |  | SpbK | 1 | 203 | 206 |
|  | 2 | 153 |  | Hna | 1 | 102 | 102 | PD-T7-4 | 1 | 206 | 209 |  | 2 | 3 |  |
|  | 3 | 26 |  | Kiwa | 1 | 106 | 109 |  | 2 | 3 |  | SspABCDE | 1 | 2 | 2 |
|  | 4 | 5 |  |  | 2 | 3 |  | PD-T7-5_1 | 1 | 68 | 72 | Stk2 | 1 | 164 | 164 |
|  | 5 | 1 |  | Lamassu | 1 | 871 | 991 |  | 2 | 4 |  | Thoeris | 1 | 457 | 501 |
| CRISPR-Cas | 1 | 685 | 1033 |  | 2 | 108 |  | PifA | 1 | 43 | 43 |  | 2 | 36 |  |
|  | 2 | 226 |  |  | 3 | 11 |  | PrrC | 1 | 10 | 84 |  | 3 | 7 |  |
|  | 3 | 109 |  |  | 4 | 1 |  |  | 2 | 74 |  |  | 4 | 1 |  |
|  | 4 | 12 |  | Lit | 1 | 6 | 6 | PsyrTA | 1 | 15 | 15 | Tiamat | 1 | 74 | 74 |
|  | 8 | 1 |  | MADS | 1 | 18 | 18 | Pycsar | 1 | 174 | 230 | Uzume | 1 | 444 | 455 |
| DarTG | 1 | 707 | 709 | MazEF | 1 | 31 | 31 |  | 2 | 35 |  |  | 2 | 11 |  |
|  | 2 | 2 |  | Menshen | 1 | 67 | 72 |  | 3 | 14 |  | Viperins | 1 | 128 | 129 |
| Dazbog | 1 | 29 | 29 |  | 2 | 1 |  |  | 4 | 7 |  |  | 2 | 1 |  |
| Detocs | 1 | 36 | 36 |  | 3 | 4 |  | Retron | 1 | 166 | 197 | VSPR | 1 | 7 | 7 |
| DISARM | 1 | 125 | 128 | Mokosh | 1 | 5314 | 5962 |  | 2 | 31 |  | Wadjet | 1 | 408 | 494 |
|  | 2 | 3 |  |  | 2 | 637 |  | RloC | 1 | 75 | 76 |  | 2 | 78 |  |
| Dnd | 1 | 389 | 521 |  | 3 | 11 |  |  | 2 | 1 |  |  | 3 | 4 |  |
|  | 2 | 23 |  | Nhi | 1 | 754 | 757 |  |  |  |  |  | 4 | 4 |  |
|  | 3 | 108 |  |  | 2 | 3 |  |  |  |  |  |  |  |  |  |
|  | 4 | 1 |  | NixI | 1 | 3 | 3 |  |  |  |  |  |  |  |  |
|  |  |  |  |  |  |  |  |  |  |  |  |  |  |  |  |
|  |  |  |  |  |  |  |  |  |  |  |  |  |  |  |  |

**Supplementary Table S2. Summary of all CRISPR-Cas systems validated in the *Bacillus cereus* group.** Prevalence calculated as the sum of assemblies^(*1)^ / Total count of assemblies^(*2)^ *100.

| **GTDB Species** | **Minimum per genomic assembly** | **Maximum per genomic assembly** | **Mean** | **Median** | **Sum of occurrences** | **Sum of assemblies where the system is present^(*1)^** | **Total count of assemblies^(*2)^** | **Prevalence (%)** |
| --- | --- | --- | --- | --- | --- | --- | --- | --- |
| *Bacillus albus* | 1 | 1 | 1 | 1 | 2 | 2 | 26 | 7.69 |
| *Bacillus anthracis* | 1 | 1 | 1 | 1 | 2 | 2 | 766 | 0.26 |
| *Bacillus cereus* | 1 | 2 | 1.04 | 1 | 304 | 291 | 2145 | 13.57 |
| *Bacillus cytotoxicus* | 1 | 3 | 1.86 | 2 | 78 | 42 | 43 | 97.67 |
| *Bacillus mobilis* | 1 | 1 | 1 | 1 | 1 | 1 | 44 | 2.27 |
| *Bacillus mycoides* | 1 | 2 | 1.11 | 1 | 30 | 27 | 168 | 16.07 |
| *Bacillus paramycoides* | 1 | 1 | 1 | 1 | 1 | 1 | 16 | 6.25 |
| *Bacillus pseudomycoides* | 1 | 2 | 1.03 | 1 | 37 | 36 | 118 | 30.51 |
| *Bacillus thuringiensis* | 1 | 2 | 1.01 | 1 | 301 | 297 | 1202 | 24.71 |
| *Bacillus toyonensis* | 1 | 2 | 1.02 | 1 | 42 | 41 | 314 | 13.06 |
| *Bacillus wiedmannii* | 1 | 1 | 1 | 1 | 8 | 8 | 209 | 3.83 |
| Other | 1 | 1 | 1 | 1 | 7 | 7 | 238 | 2.94 |
